# Identification of Multipotent Progenitors that Emerge Prior to Hematopoietic Stem Cells in Embryonic Development

**DOI:** 10.1016/j.stemcr.2014.02.001

**Published:** 2014-03-20

**Authors:** Matthew A. Inlay, Thomas Serwold, Adriane Mosley, John W. Fathman, Ivan K. Dimov, Jun Seita, Irving L. Weissman

**Affiliations:** 1 Institute for Stem Cell Biology and Regenerative Medicine (ISCBRM), Stanford University, Stanford, CA 94305, USA; 2 Joslin Diabetes Center, Harvard Medical School, Boston, MA 02215, USA; 3 Genomics Institute of the Novartis Research Foundation, San Diego, CA 92121, USA; 4 Ludwig Center for Cancer Stem Cell Research and Medicine, Stanford University School of Medicine, Stanford, CA 94305, USA

## Abstract

Hematopoiesis in the embryo proceeds in a series of waves, with primitive erythroid-biased waves succeeded by definitive waves, within which the properties of hematopoietic stem cells (multilineage potential, self-renewal, and engraftability) gradually arise. Whereas self-renewal and engraftability have previously been examined in the embryo, multipotency has not been thoroughly addressed, especially at the single-cell level or within well-defined populations. To identify when and where clonal multilineage potential arises during embryogenesis, we developed a single-cell multipotency assay. We find that, during the initiation of definitive hematopoiesis in the embryo, a defined population of multipotent, engraftable progenitors emerges that is much more abundant within the yolk sac (YS) than the aorta-gonad-mesonephros (AGM) or fetal liver. These experiments indicate that multipotent cells appear in concert within both the YS and AGM and strongly implicate YS-derived progenitors as contributors to definitive hematopoiesis.

## Highlights

• A multipotency assay reveals eight hematopoietic lineages from a single cell
• The first embryonic multipotent cells are defined as CD11A^−^ KIT^+^ SCA-1^+^ (CD11A^−^ KLS)
• Transplanted CD11A− KLS cells give rise to robust hematopoiesis in newborn mice
• The early yolk sac is implicated as the major source of CD11A^−^ KLS cells

## Teaser

The embryonic precursors that give rise to hematopoietic stem cells (HSCs) remain undefined. Here, Weissman, Inlay, and colleagues identify the earliest arising embryonic progenitors that can differentiate into all major hematopoietic lineages in vitro and in vivo. These progenitors are present within the embryo prior to HSC emergence but are most abundant within the extraembryonic yolk sac and may be precursors to HSCs.

## Introduction

In the mammalian blood system, all mature blood lineages, including erythrocytes, platelets, and all innate and adaptive immune cells, are generated from hematopoietic stem cells (HSCs). In adults, HSCs reside almost exclusively in the bone marrow. In the embryo, however, hematopoiesis is characterized by distinct yet overlapping waves of blood development, appearing in multiple sites, with primitive erythroid-biased waves succeeded by definitive waves with increasing lineage potential and functionality. The functional properties that define adult HSCs do not appear at once during development but emerge gradually over the course of several days.

In the mouse embryo, the first blood-forming cells appear approximately 7.5 days into gestation (embryonic day [E] 7.5) within the blood islands that line the extraembryonic yolk sac (YS) (Moore and Metcalf, 1970). These “primitive” blood-forming cells appear to be lineage-restricted, form primarily large nucleated erythrocytes, and express embryonic globins (Palis et al., 1999). They also lack the ability to engraft when transplanted intravenously into lethally irradiated adult mice, a hallmark property of fully functional adult bone marrow HSCs (Müller et al., 1994). After the establishment of a circulatory system at e8.5, “definitive” erythromyeloid progenitors appear within the YS (Palis et al., 1999), the placenta (PL) (Alvarez-Silva et al., 2003), and the embryo proper (EP). The earliest intraembryonic hematopoietic progenitors are found within the para-aortic splanchnopleura (p-Sp), which develops into the aorta-gonad-mesonephros (AGM) that contains the dorsal aorta (Cumano et al., 1996, Godin et al., 1993, Godin et al., 1995, Medvinsky et al., 1993). Hematopoietic progenitors with the ability to self-renew appear within the YS and AGM at e9.0 and appear within the fetal liver (FL) a day or two later (Yoder and Hiatt, 1997). e9.5 YS cells lack the ability to home to the bone marrow when transplanted into adult mice, but their long-term self-renewal activity can be revealed in vivo by transplantation into the liver or facial vein of sublethally irradiated newborn mice (Yoder and Hiatt, 1997, Yoder et al., 1997a, Yoder et al., 1997b) or alternatively by first coculturing with reaggregated AGM tissue (Taoudi et al., 2008) or on the OP9 bone marrow stromal line (Rybtsov et al., 2011), indicating that progenitors residing within the YS can mature into functional HSC. These embryonic progenitors were thought to be precursors to HSCs, or “pre-HSCs,” and whereas not precisely defined, pre-HSCs expressed markers associated with endothelial (VE-cadherin) and hematopoietic (CD41 then CD45) cells (Rybtsov et al., 2011). At e10.5, fully functional HSCs have been isolated from the dorsal aorta of the AGM region (Müller et al., 1994), the extraembryonic YS, PL (Gekas et al., 2005), and from the vitelline and umbilical vessels (de Bruijn et al., 2000). At e11.5, HSCs are also found within the FL, which then becomes the predominant site of hematopoiesis until the formation of a bone-marrow cavity several days later (Gekas et al., 2005, Müller et al., 1994). Thus, the maturation of blood-forming cells takes place in discrete steps and likely at several different sites.

A fundamental unresolved question is whether definitive hematopoietic cells derive directly from the primitive precursors that first appear in the YS blood islands (Moore and Metcalf, 1970) or instead emerge separately from a hematoendothelial precursor in the dorsal aorta called hemogenic endothelium (Dzierzak and Medvinsky, 1995, Nishikawa et al., 1998). A large body of evidence supports the de novo generation of HSCs within the dorsal aorta, including ex vivo tissue explants of the dorsal aorta prior to circulation (Cumano et al., 1996, Cumano et al., 2001, Medvinsky and Dzierzak, 1996). Also, time-lapse imaging of AGM sections in culture reveals the emergence of hematopoietic clusters from within the luminal wall of the dorsal aorta in mice, which express several HSC markers, such as KIT, SCA-1, and CD41 (Boisset et al., 2010). Definitive hematopoietic progenitors also exist within the YS (Huang and Auerbach, 1993, Kumaravelu et al., 2002). However, early studies could not exclude the possibility that such progenitors originated elsewhere and then migrated to the YS. Evidence supporting a distinct YS origin of definitive hematopoiesis comes from lineage-tracing experiments that used a *Runx1* Cre-estrogen receptor (ER) reporter to exclusively label YS-derived hematopoietic cells; subsequent analysis of these mice revealed labeling of adult HSCs (Samokhvalov et al., 2007). Similarly, inducible rescue of *Runx1* expression in *Runx1* knockout embryos demonstrated that definitive hematopoiesis could only be rescued at the developmental stages when Runx1 expression was restricted to the YS (Tanaka et al., 2012). In *Ncx1*^**−**/**−**^ embryos, which lack a heartbeat and thus circulation, all hematopoietic cells are found within the YS and PL prior to embryonic lethality at e10.5 (Lux et al., 2008, Rhodes et al., 2008). Additionally, transplantation of YS cells from e8 to e9 allogeneic donors into the YS cavities of e8 to e9 hosts in utero led to YS blood-island engraftment and, when analyzed several months after birth, gave rise to donor-derived spleen colony-forming myeloerythroid cells and thymic and peripheral T cells (Weissman et al., 1977, Weissman et al., 1978). Therefore, maturation of early YS stem/progenitors to adult HSC was demonstrated, but the cellular identity of HSC precursors, their sites of maturation, and the molecular mechanisms involved remain a mystery.

Identification of the key populations that give rise to each wave of embryonic hematopoiesis may provide critical insights into the relationship between primitive and definitive hematopoiesis. However, the surface markers used to isolate adult HSCs and downstream stages have proven unreliable for identifying the equivalent embryonic populations (Cumano and Godin, 2007). As a result, the cells that initiate each hematopoietic wave remain poorly defined. Multipotency in hematopoiesis refers to the ability of a progenitor to give rise to all blood lineages: myeloid cells including erythrocytes, platelets, monocytes, tissue macrophages, and granulocytes, as well as lymphocyte lineages including T, B, natural killer (NK), and dendritic cells. Multipotency also distinguishes definitive hematopoiesis from more primitive cells with limited lineage potential. Conclusive evidence of multipotency requires a single-cell assay to prevent the false positives that may occur when mixtures of lineage-committed progenitors collectively produce all lineages. In the embryo, multipotency was first observed within the AA4.1^**+**^ p-Sp population at e9.5 (Godin et al., 1995). YS AA4.1^**+**^ WGA^hi^ cells possess myeloid and lymphoid potential in vitro by e10.5 and robustly by e11.5, though this was not demonstrated clonally (Huang and Auerbach, 1993). Thus, whereas much is known about the timing of appearance of progenitor potentials within the early embryo, the clonal identities of the cells with multipotent potentials are less clear. Therefore, we sought to define the earliest clonally multipotent cells and to determine their distribution within the sites of early hematopoiesis.

## Results

### Development of an In Vitro Clonal Multilineage Assay

**Figure 1:**
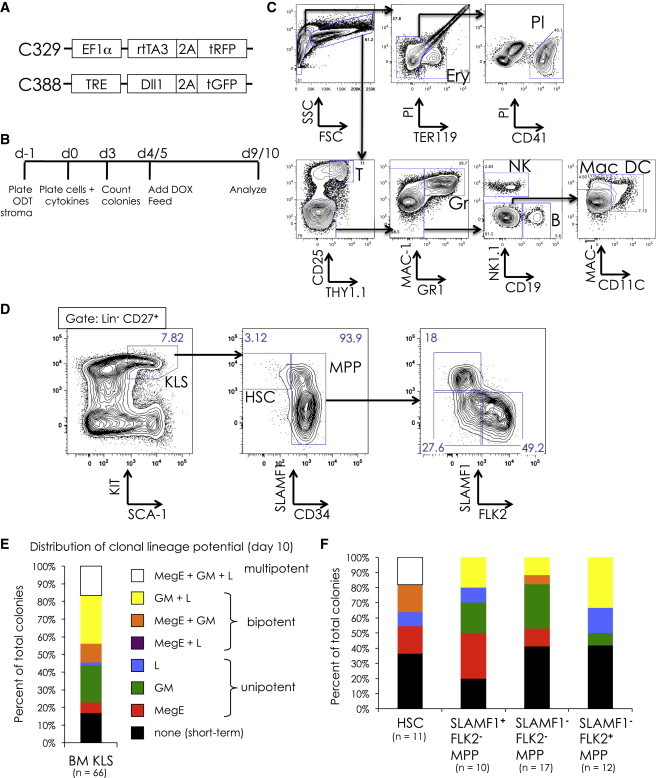
Development of an In Vitro Clonal Multipotency Assay (A) Lentiviral constructs used to generate a tetracycline-inducible *Dll1* OP9 stromal line (ODT). Construct C329 (top) drives constitutive expression of the tetracycline-inducible transactivator rtTA3. Construct C388 allows induction of *Dll1* expression when the transactivator is activated in the presence of doxycycline (DOX). (B) Strategy for clonal assay. ODT stroma is plated the day prior to cell sorting (day −1). At day 0, cells are clone sorted directly onto ODT stroma and cytokines are added (SCF, TPO, EPO, Flt3L, IL-7, and IL-15). At day 3, hematopoietic colonies are counted. At day 5, cells are fed and Flt3L, IL-7, IL-15, and DOX (1 μg/ml) are added. At day 9 or 10, colonies are harvested and analyzed by FACS. (C) Representative hematopoietic output at day 10 of culture of unsorted e12.5 FL. Representative examples of multipotent output from single-cell cultures can be found in Figure S1. (D–F) Testing multipotency assay on adult bone marrow (BM) stem/progenitor cells. (D) Sort gates for KIT^**+**^ Lin^**−**^ SCA-1^**+**^ (KLS) cells and subpopulations of KLS, including HSCs (CD34^**−**^, SLAMF1^**+**^) and three fractions of multipotent progenitors (MPP) are shown. (E) The distribution of lineage potential of colonies from adult BM KLS cells, showing cells that produced a single branch (MegE in red, GM in green, and L in blue), two branches (MegE + GM in orange, GM + L in yellow, and MegE + L in purple), and all three branches (MegE + GM + L in white). Cells that produced lineages from all three branches (white) were scored as multipotent. Cells that gave rise to colonies that did not survive to day 10 are shown in black. The number of hematopoietic colonies scored (n) is indicated. (F) Distribution of lineage potential in colonies derived from sorted KLS subpopulations. Note that only HSCs gave multipotent (white) readout.

In order to identify clonally multipotent populations throughout embryonic development, we developed a single-cell multipotency assay (Figure 1). The bone-marrow-derived OP9 stromal line can be used to generate most hematopoietic lineages in culture, with the notable exception of T lymphocytes (Kodama et al., 1994). Alternatively, a modified OP9 stromal line that expresses *Dll1* (OP9-DL1) can promote T lineage development but inhibits B cell development (Schmitt and Zúñiga-Pflücker, 2002). Because of the importance of detecting lymphocyte potential in distinguishing definitive hematopoietic cells from their primitive myeloid-restricted counterparts, we sought a method to generate both B cells and T cells from a single cell within a single well. We previously published an assay whereby common lymphocyte progenitors could be cultured and expanded on OP9 stroma without committing to either the B or T cell lineages (Karsunky et al., 2008). We therefore generated an OP9 stromal line with an inducible *Dll1* expression cassette (Figure 1A). In this line, which we call ODT, addition of doxycycline (DOX) rapidly induces surface expression of *Dll1*, driving uncommitted lymphocyte progenitors to the T cell lineage, whereas B-committed progenitors resist T cell induction and continue to produce B cells (M.A.I. and I.L.W., unpublished data). Hematopoietic progenitors were plated on ODT stroma along with a combination of hematopoietic cytokines (SCF, TPO, EPO, Flt3L, interleukin [IL]-7, and IL-15); DOX was added 4 to 5 days into the culture (Figure 1B), leading to eight hematopoietic lineages (erythrocytes, platelets, macrophages, granulocytes, dendritic cells, natural killer cells, B cells, and T cells), representing the three major branches of hematopoiesis: megakaryocyte/erythrocyte (MegE), granulocyte/monocyte (GM), and lymphoid (L) (Figure 1C). Representative fluorescence-activated cell sorting (FACS) plots of single-cell-derived colonies scored as multipotent can be found in Figure S1 available online. To validate this assay, we sorted adult bone marrow (BM) KIT^**+**^ Lin^**−**^ SCA-1^**+**^ (KLS) cells, a population that contains HSCs and multipotent progenitors, and analyzed their clonal lineage potential (Figures 1D and 1E). Individual KLS cells produced a variety of different lineage outcomes: some cells yielded only a single lineage, others generated multiple lineages, and about 15% of colonies produced lineages representing each of the three major hematopoietic branches. These colonies we scored as multipotent. We next separated the KLS population into four subsets based on expression of CD34, SLAMF1, and FLK2, markers that identify HSCs and other multipotent progenitor populations, and repeated the assay (Figures 1D and 1F). Whereas each population could collectively produce all lineages, we found that only the HSC population (CD34^**−**^ FLK2^**−**^ SLAMF1^**+**^ KLS) contained multipotent cells, despite the fact that all KLS subpopulations are known to be multipotent in vivo. Thus, this assay can reveal multipotency of individual cells, although not all multipotent cells are revealed.

### Nearly All Hematopoietic Colony-Forming Cells Reside in the KIT^+^ CD43^+^ Fraction

In our assay conditions, hematopoietic stem and progenitor cells give rise to distinct colonies by day 3, and thus we could also use this assay to identify markers for these cells. We initially screened eight markers (KIT, CD43, CD34, CD41, SCA-1, CD45, AA4.1, and SLAMF1) from YS, AGM, and FL tissues from e9.5 to e12.5 to identify which markers could consistently enrich or deplete for colony-forming activity (Figure S2). For most markers, colony-forming activity was found in both the positive and negative fractions. For example, whereas the hematopoietic marker CD41 could enrich hematopoietic colony-forming ability at e9.5, it became downregulated at later time points, resulting in colony-forming activity within the CD41^**−**^ fraction (Figure S2A). However, two markers, KIT and CD43, nearly uniformly marked all colony-forming cells in all tissues from e9.5 to e12.5.

### Emergence of Multipotent KLS Cells during Embryogenesis

**Figure 2:**
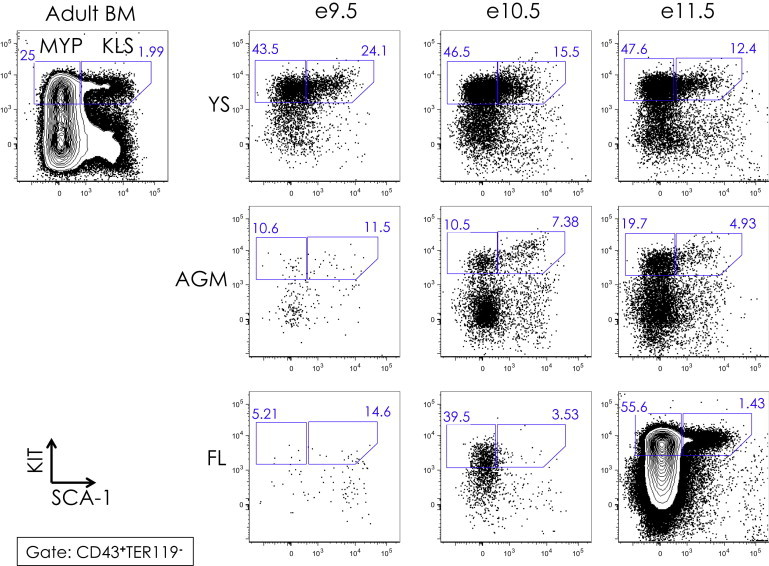
KIT versus SCA-1 Expression of CD43^**+**^ Cells in YS, AGM, and FL from e9.5 to e11.5 KIT (y axis) and SCA-1 (x axis) expression of CD43^**+**^ adult BM is shown in the upper left corner. Gates of KIT^**+**^ SCA-1^**+**^ “KLS” and KIT^**+**^ SCA-1^**−**^ myeloid progenitors “MYP” are shown, with the percent of KLS and MYP populations among CD43^**+**^ TER119^**−**^ cells indicated. See Figure S3A for CD43/TER119 plots and gates.

Based on our initial screen, we restricted the search for multipotent cells to subpopulations within the KIT^**+**^ CD43^**+**^ fraction. We stained tissues with a variety of antibodies to identify candidate populations within the KIT^**+**^ CD43^**+**^ fraction that we could test for clonal multilineage potential (Figures 2 and S3). In the adult, all multipotent stem and progenitors are found in the KLS fraction. We identified a similar population that is KIT^**+**^ CD43^**+**^ and SCA-1^**+**^, which appears in e9.5 YS and AGM and e11.5 FL (Figure 2). This population is highly enriched for colony-forming activity (between 35% and 50% of plated cells gave rise to colonies) and, as a population, gives rise to all lineages in vitro (data not shown). We refer to this embryonic population as KLS (KIT^**+**^ Lin^**−**^ SCA-1^**+**^) to reflect its similarity to the analogous KLS population in adult BM, although embryonic KLS cells also are defined by expression of CD43, and the only required lineage marker for negative gating is the red-blood-cell marker TER119 (Figure S3A).

In adult BM, myeloid progenitors are found within the KIT^**+**^ SCA-1^**−**^ (MYP) fraction, which contains common myeloid progenitors (CMP), granulocyte-monocyte progenitors (GMP), and megakaryocyte-erythrocyte progenitors (MEP) (Akashi et al., 2000). In the embryo, we identified a similar MYP population, from which emerged CMP and then MEP and GMP, both by surface phenotype and in vitro lineage potential (Figure S3B; data not shown).

### CD11A Expression Divides Embryonic KLS into Two Distinct Fractions

**Figure 3:**
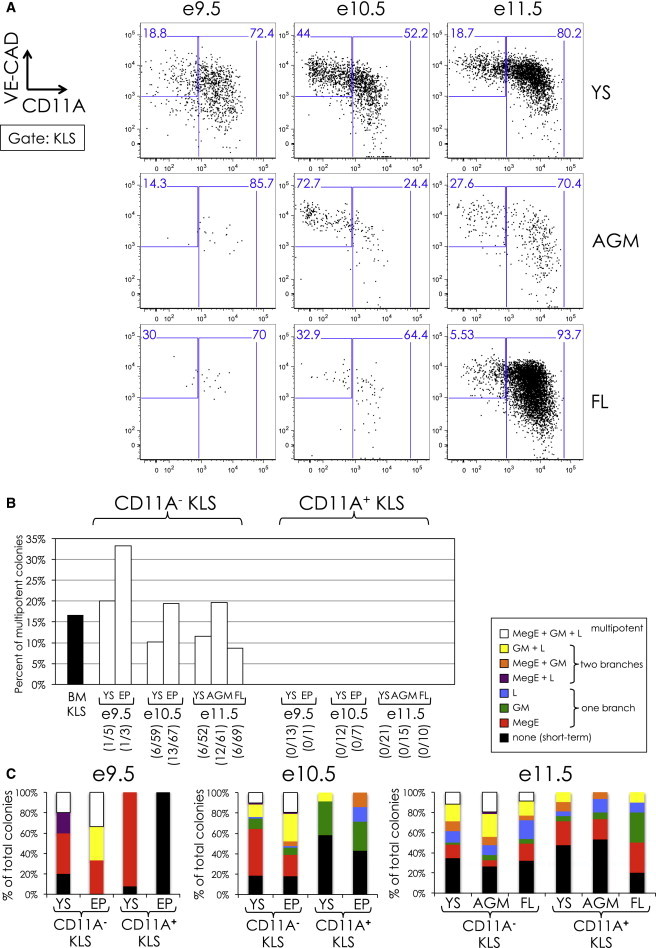
Clonal Analysis of CD11A^**−**^ and CD11A^**+**^ KLS (A) VE-cadherin (VE-CAD; y axis) versus CD11A (x axis) expression of embryonic KLS from e9.5 to e11.5. (B) Percent of multipotent colonies from clone-sorted CD11A^**−**^ and CD11A^**+**^ KLS subsets. The percent of multipotent colonies arising from adult BM KLS is indicated in black and was duplicated from Figure 1E. For e9.5 and e10.5, CD11A^**−**^ and CD11A^**+**^ KLS cells derived from the entire embryo proper (EP) were analyzed. The number of multipotent colonies observed out of total colonies scored is listed in parentheses. (C) Lineage distribution of CD11A^**−**^ and CD11A^**+**^ KLS colonies. The distribution of lineages that resulted from clone-sorted CD11A^**−**^ and CD11A^**+**^ KLS cells onto ODT stroma from e9.5 to e11.5 is shown. A description of the scoring system can be found in the legend to Figure 1E. The percentage of colonies scored as multipotent is shown as white bars and is identical to the data shown in (B).

The surface markers SLAMF1, FLK2, and CD34 are known to subdivide adult KLS into HSCs and downstream multipotent progenitors (Figure 1D). We examined these markers in embryonic KLS and found that their expression levels varied from time point to time point and from tissue to tissue, making the use of these markers unreliable to subdivide embryonic KLS prior to e11.5 (Figure S4). However, we did find that the KLS population could be consistently subdivided based on expression of CD11A (Figure 3A). CD11A (*Itgal*) is a component of the leukocyte adhesion complex lymphocyte function associated 1 (LFA-1), an integrin involved in several immune system functions including leukocyte trafficking and lymphocyte activation (Hibbs et al., 1991, Hogg et al., 2011). In a related manuscript, we show that adult BM HSCs can also be subdivided based on CD11A expression and only the CD11A^**−**^ fraction contains functional HSCs (J.W.F., M.A.I., Nathaniel B. Fernhoff, J.S., and I.L.W., unpublished data). CD11A^**−**^ KLS cells uniformly expressed high levels of the endothelial adhesion molecule VE-cadherin (VE-CAD), whereas CD11A^**+**^ KLS cells expressed variable levels of VE-cadherin (Figure 3A). We also examined the expression of several other markers including the endothelial-associated markers TIE2 and endoglin and the hematopoietic markers CD45 and MAC-1 (Figure S5). Whereas the expression patterns of these markers were less consistent than CD11A and VE-cadherin across all tissues and time points, in general we found that endothelial markers were more highly expressed at earlier time points and on CD11A^**−**^ KLS cells, whereas hematopoietic markers were more highly expressed at later time points and on CD11A^**+**^ KLS cells.

We next tested CD11A^**−**^ and CD11A^**+**^ KLS cells for clonal multilineage potential (Figures 3B and 3C). In adult BM, the KLS population contains all multipotent cells (Figures 1D–1F). At the population level, both CD11A^**−**^ and CD11A^**+**^ KLS cells were able to give rise to all lineages, including lymphoid B, T, and NK cells (data not shown). However, only the CD11A^**−**^ fraction was able to simultaneously give rise to MegE, GM, and lymphoid cells at the single-cell level, indicating the presence of multipotent cells within this population (Figures 3B and 3C). We found multipotent CD11A^**−**^ KLS cells in all tissues examined, from e9.5 to e11.5. Conversely, in the CD11A^**+**^ KLS population, we found bipotent MegE/GM and GM/L colonies but never all three branches at once, regardless of time point or tissue (Figure 3C). Thus, the CD11A^**−**^ KLS cells population contains all the multipotent cells from e9.5 to e11.5 and appears in both extra- and intraembryonic regions around e9.5.

### YS Contains the Most Multipotent Cells from E9.5 to E11.5

**Figure 4:**
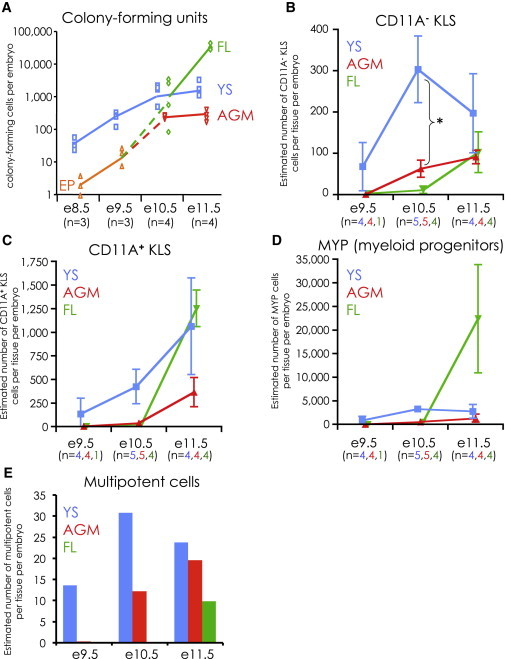
Numeric Analysis of Hematopoietic Progenitor Cells in Embryonic Development (A) Absolute number of colony-forming cells per embryo from YS (blue squares), AGM (red downward triangles), and FL (green diamonds) from e8.5 to e11.5. At e8.5 and e9.5, the whole embryo proper (EP; orange upright triangles) was cultured instead of AGM and FL. Unsorted tissues were plated onto ODT stroma at serial dilutions and colonies counted at day 3. The number of litters analyzed (n) for each time point is indicated. (B–D) The absolute numbers of CD11A^**−**^ KLS (B), CD11A^**+**^ KLS (C), and MYP (D) cells were calculated for YS (blue), AGM (red), and FL (green) from e9.5, e10.5, and e11.5. The numbers shown are per tissue, per embryo. Error bars are SD. The asterisk (^∗^) in (B) indicates a statistically significant difference (unpaired t test) between the absolute number of CD11A^**−**^ KLS cells in the YS and AGM at e10.5 (p < 0.002). The number of litters analyzed (n) for each tissue (YS in blue, AGM in red, FL in green) is indicated. (E) Estimation of the absolute number of clonally multipotent cells per tissue per embryo. These numbers were generated by multiplying the absolute number of CD11A^**−**^ KLS (from B) times the percent of multipotent cells contained within (from Figure 3B).

Our assay also allowed us to estimate the numbers of multipotent progenitors in each tissue from e9.5 to e11.5, based on colony-forming activity (Figure 4A). We found that, from e8.5 to e10.5, the YS contained the majority of progenitors, peaking around 1,000 colony-forming cells at e10.5 and e11.5. By e11.5, the FL had surpassed the YS and became the predominant source of hematopoiesis from then on. At all time points examined, the AGM contained the fewest colony-forming cells.

We also estimated the absolute number of CD11A^**−**^ KLS, CD11A^**+**^ KLS, and MYP (Lin^**−**^ CD43^**+**^ KIT^**+**^ SCA-1^**−**^) populations in each tissue (Figures 4B–D). Collectively, these three populations contain all KIT^**+**^ CD43^**+**^ cells and, thus, likely all hematopoietic stem/progenitors. We found that the YS contained approximately 300 CD11A^**−**^ KLS cells per embryo at e10.5, roughly six times as many as in the AGM at this time point (Figure 4B). Furthermore, CD11A^**−**^ KLS numbers appeared to decrease in the YS at e11.5 and increase in the AGM and FL. At e11.5, the FL contained the most CD11A^**+**^ KLS and MYP (Figures 4C and 4D). Because CD11A^**−**^ KLS cells contain all multipotent activity at e11.5, this suggests that the bulk of the cells that initially seed the FL are CD11A^**+**^ KLS and MYP, which we hypothesize are downstream of CD11A^**−**^ KLS.

With our estimates of the absolute number of CD11A^**−**^ KLS cells (Figure 4B) and the frequency of multipotent cells within each tissue (Figure 3B), we could estimate the absolute number of multipotent cells (Figure 4E). We found that e10.5 YS contains the most multipotent cells of all tissues and time points examined, and at e11.5, the YS still contained more multipotent cells than AGM or FL. Taken together, our examination of the absolute number of CD11A^**−**^ KLS and multipotent cells suggests that the YS is a major source of multipotent cells at the stages when fully functional HSCs first appear in embryonic development.

### EPCR Marks a Subset of CD11A^−^ KLS and Can Partially Enrich for Multipotency

**Figure 5:**
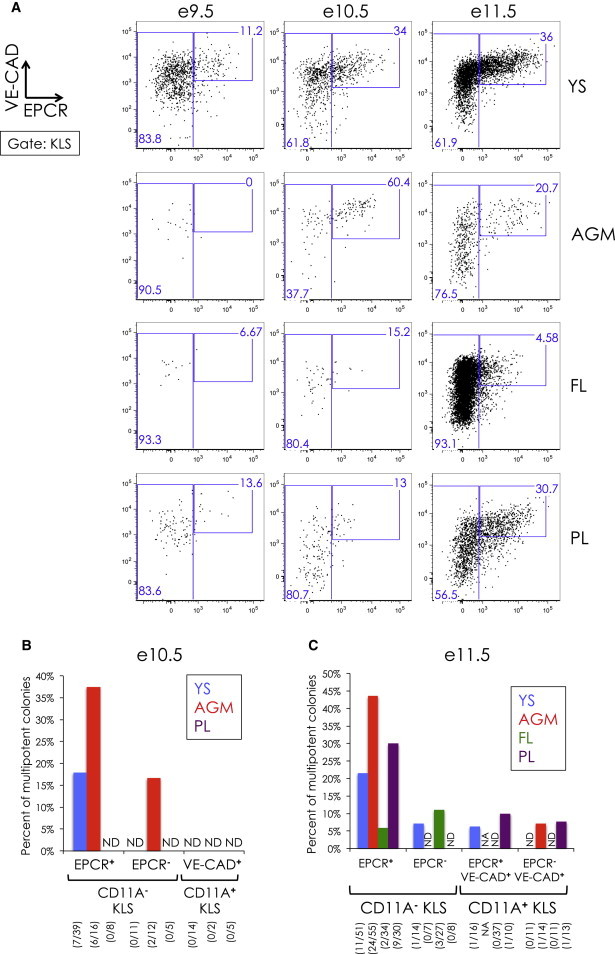
Clonal Multipotent Analysis of EPCR^**+**^ and EPCR^**−**^ KLS Subsets (A) VE-CAD (y axis) versus EPCR (x axis) expression of KLS cells in YS, AGM, FL, and PL from e9.5 to e11.5. Gates and percentages of EPCR^**+**^ and EPCR^**−**^ KLS cells are indicated. Nearly all EPCR^**+**^ cells were CD11A^**−**^ (data not shown). (B and C) Percent of multipotent colonies in EPCR^**+**^ and EPCR^**−**^ KLS subsets in e10.5 (B) and e11.5 (C) CD11A^**−**^ and CD11A^**+**^ KLS cells. For CD11A^**+**^ KLS, only VE-CAD^**+**^ cells were analyzed. ND, not detected; NA, not analyzed. The number of multipotent colonies observed out of total colonies scored is indicated in parentheses. FACS analyses of representative multipotent colonies from e11.5 EPCR^**+**^ CD11A^**−**^ KLS cells can be found in Figure S1.

The protein C receptor EPCR (CD201; *Procr*) has previously been reported to be expressed in adult HSCs, as well as embryonic FL e12.5 HSCs (Balazs et al., 2006, Iwasaki et al., 2010). We found that EPCR is expressed on a subset of KLS cells as early as e9.5 and remains expressed throughout gestation (Figure 5A). EPCR expression correlates with high VE-CAD expression and lower CD11A expression. Using the clonal multipotency assay, we observed multipotent colony formation from both EPCR^**+**^ and EPCR^**−**^ subsets of CD11A^**−**^ KLS cells, though multipotency appeared greater in the EPCR^**+**^ subfraction overall (Figures 5B, 5C, and S1). We also identified CD11A^**−**^ and CD11A^**+**^ KLS cells in the placenta (Figure S6), a region known to be a niche for hematopoietic stem cells (Gekas et al., 2005, Ottersbach and Dzierzak, 2005), and included placental populations in the analysis. Although we did not observe multipotency in e10.5 PL CD11A^**−**^ KLS, we did find it within the EPCR^**+**^ CD11A^**−**^ KLS at e11.5 in the PL. Taken together, our data suggest that EPCR can partially enrich for multipotent cells, though not all multipotent cells are EPCR^**+**^.

### Lineage-Tracing KLS Cells In Vivo

To better understand the lineage relationship between CD11A^**−**^ and CD11A^**+**^ KLS cells, we examined two lineage-tracing reporter mouse strains (Figure S7). CD11A^**−**^ KLS cells generally express higher levels of TIE2 than CD11a^**+**^ KLS cells (Figure S5A); thus, we crossed Tie2^Cre^ mice (Kisanuki et al., 2001) to the mT/mG reporter strain (Muzumdar et al., 2007), which permanently switches from Tomato to GFP fluorescence in any cell that expresses Cre (Figure S7A). In the Tie2^cre^ × mT/mG embryos, we found that all KLS cells were GFP^+^ and thus derived from TIE2-expressing precursors (Figure S7B). However, other cell types express TIE2, including hemogenic endothelium, so we next crossed VE-cadherin^CreER^ mice (Monvoisin et al., 2006, Zovein et al., 2008) to mT/mG reporters (Figures S7C–S7E). In this cross, when tamoxifen is administered, all cells that express VE-cadherin during the 24 hr window when tamoxifen is active will permanently express GFP in themselves and in their progeny. Because VE-cadherin expression is associated with earlier time points and more multipotent cells, we hypothesized that VE-CAD^**+**^ cells would precede VE-CAD^**−**^ cells. We injected tamoxifen into pregnant females and examined embryos at 24, 48, and 72 hr after injection to monitor the distribution of GFP-expressing KLS cells. We found that, at 24 hr, the majority of labeled KLS cells were VE-CAD^**+**^ (both CD11A^**−**^ and CD11A^**+**^), but at 48 and 72 hr, the fraction of labeled VE-CAD^**−**^ KLS cells increased and the frequency of labeled CD11A^**−**^ KLS cells decreased, consistent with the notion that VE-CAD^**+**^ KLS cells are giving rise to VE-CAD^**−**^ KLS cells. Whereas neither reporter conclusively demonstrates that CD11A^**−**^ KLS cells give rise to CD11A^**+**^ KLS cells, our data support our contention that early hematopoietic progenitors that express endothelial-associated markers generally precede those that express hematopoietic markers.

### Multipotency Moves into the CD11A^+^ KLS Subfraction at E12.5

**Figure 6:**
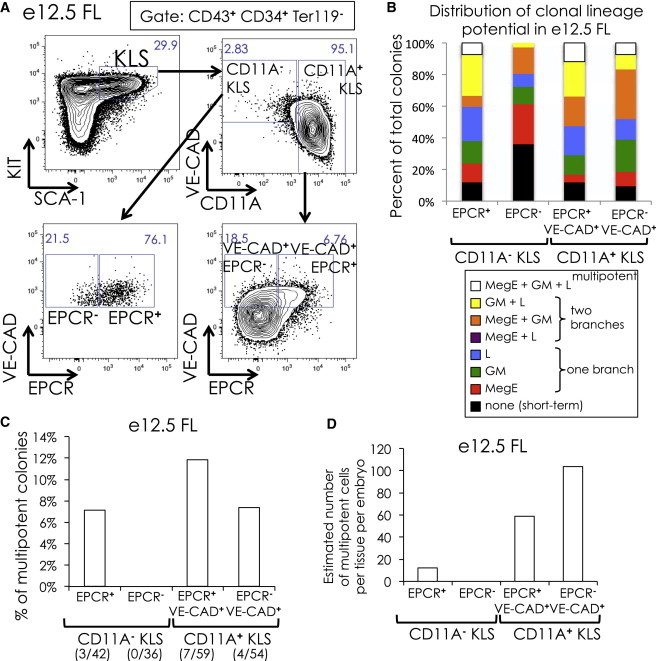
Clonal Analysis of E12.5 FL KLS Subsets (A) Gating of CD11A^**−**^ and CD11A^**+**^ KLS cells in e12.5 FL. Only CD43^**+**^ CD34^**+**^ TER119^**−**^ cells are shown. Percent of cells within each gate are shown. (B) Distribution of clonal lineage potential in e12.5 FL EPCR^**+**^ and EPCR^**−**^ CD11A^**−**^ and CD11A^**+**^ KLS cells. The legend is described in Figure 1E. Only CD11A^**−**^ KLS and VE-CAD^**+**^ CD11A^**+**^ KLS cells were analyzed. (C) Percent of multipotent colonies in e12.5 FL CD11A^**−**^ and CD11A^**+**^ KLS subsets. The number of multipotent colonies observed out of total colonies scored is indicated in parentheses. (D) Estimation of the absolute number of multipotent colonies per embryo in e12.5 FL CD11A^**−**^ and CD11A^**+**^ KLS cells, calculated as in Figure 4E.

By e12.5, the FL is by far the dominant site of hematopoiesis and is known to produce fully functional HSCs by transplantation (Morrison et al., 1995). When we examined e12.5 FL KLS, nearly all (95%) of KLS cells were CD11A^**+**^ (Figure 6A). When we examined both CD11A^**−**^ and CD11A^**+**^ KLS fractions for clonal multilineage potential, we now found multipotency in the CD11A^**+**^ KLS fraction (Figures 6B–6D). Interestingly, only the VE-CAD^lo^ subset of CD11A^**+**^ KLS cells showed multipotency, whereas the VE-CAD^**−**^ CD11A^**+**^ KLS subset, which represents over ¾ of CD11A^**+**^ KLS cells, lacked multipotency. By e12.5, the multipotent CD11A^**−**^ KLS population was exceedingly rare compared to the robustly expanding CD11A^**+**^ KLS population (Figures 6A and 6D). Thus, it appears that the primary source of multipotent cells shifts from CD11A^**−**^ to CD11A^**+**^ KLS cells by e12.5.

### Only CD11A^−^ KLS Cells Produce All Hematopoietic Lineages In Vivo

**Figure 7:**
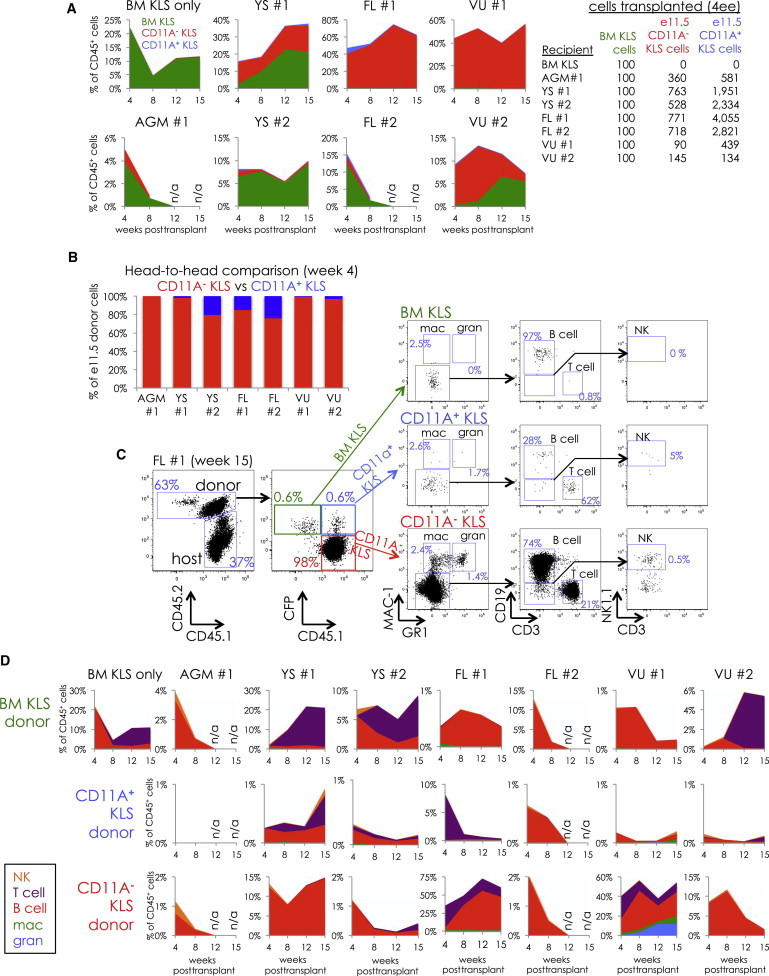
In Vivo Competitive Comparison of Engraftment and Lineage Potential of CD11A^**−**^ and CD11A^**+**^ KLS Newborn NSG mice were transplanted with four embryo equivalents of CD11A^**−**^ KLS and CD11A^**+**^ KLS cells attained from different tissues at e11.5 along with 100 adult BM KLS cells. Blood was analyzed at 4, 8, 12, and 15 weeks to examine donor chimerism and lineages produced. (A) Comparison of donor chimerism between BM KLS (green), CD11A^**−**^ KLS (red), and CD11A^**+**^ KLS (blue). Stacked graphs show the distribution of donor cells in the blood at 4, 8, 12, and 15 weeks posttransplant, listed as a percentage of total CD45^**+**^ cells (including those of the recipient). The tissue of origin for e11.5 KLS cells is indicated above each graph. Note that the scale of the y axis is different in each graph. The number of CD11A^**−**^ and CD11A^**+**^ KLS cells for each transplant is shown on the table on the right. (B) Head-to-head comparison of e11.5 donor cells at 4 weeks. Only donor contributions of e11.5 CD11A^**−**^ KLS (red) and CD11A^**+**^ KLS (blue) were compared. (C) FACS analysis of donor lineages from e11.5 FL KLS cells (“FL #1”) at 15 weeks posttransplant. The percentage displayed for each lineage is out of that donor’s total cells. For example, the percentage of NK cells shown for CD11A^**−**^ KLS cells is out of total donor CD11A^**−**^ KLS-derived cells. (D) Time course of the distribution of donor lineages derived from adult BM KLS (top row), CD11A^**+**^ KLS (middle row), and CD11A^**−**^ KLS (bottom row) in the blood of recipient mice at 4, 8, 12, and 15 weeks posttransplant. Percentages shown are out of total CD45^**+**^ cells and shown for NK cells (orange), T cells (purple), B cells (red), macrophages (green), and granulocytes (blue). The gates used to identify each lineage are shown in (C). Note that the y axis scale is different with each graph.

The in vitro multipotency assay demonstrates that, from e9.5 to e11.5, only the CD11A^**−**^ KLS population contains clonal multilineage potential. We next compared the in vivo lineage potential of CD11A^**−**^ and CD11A^**+**^ KLS cells by competitive transplantation (Figure 7). We sorted CD11A^**−**^ and CD11A^**+**^ KLS cells from cyan fluorescent protein (CFP)^**+**^ and CFP^**−**^ embryos and mixed CFP^**+**^ CD11A^**−**^ KLS cells with CFP^**−**^ CD11A^**+**^ KLS cells, and vice versa, to directly compare engraftment and lineage potential between these two populations in the same recipient animals. We also cotransplanted 100 adult BM KLS cells as an internal control. Cells were sorted from e11.5 YS, AGM, FL, and the vitelline/umbilical (VU) region, which is also known to contain pre-HSCs and HSCs at this time point (de Bruijn et al., 2000, Gordon-Keylock et al., 2013). Because e11.5 hematopoietic progenitors have poor engraftment potential when transplanted into adult animals, we instead transplanted intravenously into irradiated newborn nonobese diabetic-severe combined immunodeficiency-γc^**−**/**−**^ (NSG) recipients. When we examined donor chimerism in the blood of recipient mice, we found substantially more donor cells derived from CD11A^**−**^ KLS cells than from CD11A^**+**^ KLS cells in all recipient mice (Figures 7A and 7B), despite transplanting nearly a 5-fold excess of CD11A^**+**^ KLS cells (Figure 7A). Whereas the level of donor chimerism varied considerably from recipient to recipient, in every case, the contribution of CD11A^**+**^ KLS cells was either minor or absent, indicating these cells are much less effective at engraftment than CD11A^**−**^ KLS cells. We also examined the lineage distribution of donor-derived cells (Figures 7C and 7D). Whereas all recipients contained donor-derived lymphocytes, only two had significant donor myeloid cells (FL #1 and VU #1). We focused on the recipient “FL #1” and found that CD11A^**−**^ KLS cells produced robust B cells, T cells, NK cells, granulocytes, and macrophages (Figure 7C). Conversely, CD11A^**+**^ KLS cells produced only a modest number of B cells and T cells, which faded over time (Figures 7C and 7D). Because erythrocytes and platelets do not express CD45, a marker we used to distinguish donor and recipient cells, we were unable to determine whether CD11A^**−**^ KLS could produce these lineages, but all other major hematopoietic lineages were detectable and robust at 15 weeks posttransplantation, suggesting that CD11A^**−**^ KLS cells are multipotent both in vivo and in vitro.

## Discussion

The essential properties of adult HSCs include the capacity to maintain themselves indefinitely (self-renewal), the ability to differentiate into all hematopoietic lineages (multipotency), and the ability to circulate from the blood to niches in the bone marrow (engraftability). It remains unclear whether HSCs develop from hematopoietic-committed precursor cells that contain some, but not all, three HSC properties or instead whether HSCs arise de novo from a hematoendothelial precursor. Earlier studies had suggested that hematopoietic precursors to HSCs (i.e., pre-HSCs) exist within the fetus and that these progenitors lacked the ability to home to the bone marrow but could be matured in vitro into bona fide, bone-marrow-homing HSCs (Gordon-Keylock et al., 2013, Rybtsov et al., 2011). We have adopted an alternative strategy to identify such precursors to HSCs. We hypothesize that HSC precursors have the property of multipotency, and we developed a single-cell assay to reductively identify, quantify, and characterize multipotent cells in the early embryo. Our data indicate that multipotent cells are predominantly localized to the YS during the stages when definitive, self-renewing HSC precursors are thought to arise (e9.5–e10.5). As the surface marker phenotype of multipotent CD11A^**−**^ KLS (VE-CAD^**+**^ CD43^**+**^ KIT^**+**^ CD11A^**−**^) cells overlaps with that of pre-HSCs (VE-CAD^**+**^ CD41^**+**^ CD45^**−**^^or^^**+**^), CD11A^**−**^ KLS and pre-HSC populations likely contain the same precursor cells. The ability of CD11A^**−**^ KLS cells to engraft in newborn mice and give rise to multiple hematopoietic lineages in vivo confirms the multilineage potential of this population and strongly implicates CD11A^**−**^ KLS cells as a critical intermediate population between the earliest primitive YS progenitors and fully functional HSCs.

It remains unclear whether HSCs arise from both intraembryonic (AGM and FL) and extraembryonic (YS and PL) tissues or if they emerge from a single site and migrate elsewhere. Our absolute cell number data indicate that the YS contains by far the most multipotent cells during the critical stages during which HSCs arise. Pre-HSCs or HSCs may also emerge de novo from hemogenic endothelium in the AGM, but our data suggest that the AGM region by itself does not produce enough multipotent cells to account for all the multipotent cells in the embryo and that likely the vast majority come from the extraembryonic YS. This is consistent with previous work estimating the absolute number of HSCs during gestation, suggesting that both the YS and AGM may contribute to the FL HSC pool (Kumaravelu et al., 2002). Additionally, our previous work showing direct orthotopic synchronic transplantation of e8 to e9 YS cells showed that YS-derived cells can indeed contribute to complete and lifelong adult hematopoiesis (Weissman et al., 1978).

CD11A^**−**^ KLS cells express multiple markers of endothelial cells (EPCR, VE-cadherin, and CD34), suggesting that they are recently derived from the endothelial lineage and raising the question of whether they retain endothelial cell potential and contain hemogenic endothelium. Whereas we did not observe any endothelial cell differentiation from CD11A^**−**^ KLS cells in our in vitro cultures, we were also unable to drive robust hematopoietic colony formation from populations that should contain hemogenic endothelium (VE-CAD^**+**^ CD34^**+**^ SCA-1^**+**^ CD43^**−**^ CD41^**−**^). Thus, it is possible that the in vitro assay described here is incapable of revealing hemogenic endothelium. Whereas the CD11A^**−**^ KLS population retains surface markers of hemogenic endothelium, the defining indicator that the CD11A^**−**^ KLS population is hematopoietic is that these cells can engraft upon intravenous transplantation into neonatal mice and give rise to multiple hematopoietic lineages.

Many of the surface markers we used to define embryonic hematopoietic populations have functional roles in cell adhesion and trafficking. VE-cadherin is an endothelial adhesion molecule involved in facilitating the adherens junctions between endothelial cells (Harris and Nelson, 2010). CD11A is part of a leukocyte adhesion complex (LFA-1) known to play a role in leukocyte extravasation from the vasculature by binding to ICAMs on endothelial cells (Hogg et al., 2011). In a related study, we show that CD11A is upregulated as HSCs lose self-renewal potential and may be involved in HSC mobilization out of the bone marrow (J.W.F., M.A.I., Nathaniel B. Fernhoff, J.S., and I.L.W., unpublished data). In this study, we show that CD11A upregulation is also associated with the loss of multilineage potential and marks a subset of lineage-committed progenitors that are downstream of embryonic multipotent cells. These changes in adhesion molecule expression may play an important role in the migration of progenitors from their tissues of origin (YS, PL, and AGM) to secondary sites of hematopoiesis (FL and BM). For example, the downregulation of VE-cadherin may allow cells to detach from the endothelium and enter circulation, whereas the upregulation of CD11A may allow cells to exit circulation and seed new sites.

In this study, we created an assay to look for clonal multilineage potential and used it to identify emerging multipotent hematopoietic progenitors within the embryo. Other markers we focused on were also critical for distinguishing multipotent cells from downstream progenitors. Due to space limitations, we include an in-depth technical discussion of these markers and their use in the Supplemental Information. Our data suggest that a multipotent, self-renewing hematopoietic wave arises in the YS blood islands and appears simultaneously in multiple sites at e9.5. We confirmed the multipotency of this population in vivo. We hypothesize that this population represents a critical intermediate in the origins of definitive hematopoiesis, and armed with a panel of markers that can identify these cells with high resolution, we can now begin to dissect the critical steps in the emergence of and maturation of the first HSCs in embryonic development.

## Experimental Procedures

### Antibodies

A detailed list of all antibodies used in this study is shown in Table S1.

### Clonal Multipotency Assay

ODT stroma was cultured as described (Vodyanik and Slukvin, 2007). Briefly, ODT was plated onto gelatin-coated dishes and cultured in the presence of OP9 media (αMEM [made from powder, Invitrogen catalog No. 12000-022] in 20% serum [Omega Scientific FB-11]). ODT was passaged around 1:10 every 5 days. OP9 cells differentiate rapidly to adipocytes when confluent, so great care was taken to avoid confluence. For clonal assays, ODT was plated the day prior to use onto gelatin-coated 96-well plates (Falcon 3072), around 1,000–2,000 cells per well, in OP9 media. The day of use, the media was replaced with 100 μl of differentiation media (described in Vodyanik and Slukvin, 2007), αMEM, 10% serum, 100 μM monothioglycerol (catalog No. M6145; Sigma), 50 μg/ml ascorbic acid (catalog No. A-0278; Sigma), 100 U/ml penicillin/100 μg/ml streptomycin (catalog No. 15140; Invitrogen), and 1× GlutaMAX (catalog No. 35050; Invitrogen) and the cytokines SCF (rmSCF; Peprotech 250-03; 10 ng/ml), TPO (rmTPO; Peprotech 315-14; 10 ng/ml), EPO (rhEPO; Invitrogen PHC2054; 0.5 U/ml), Flt3L (mFlt3L; Peprotech 250-31L; 10 ng/ml), IL-7 (mIL-7; Peprotech 217-17; 10 ng/ml), and IL-15 (IL15/IL15R complex; Ebioscience 14-8152; 5 ng/ml) were added. Colonies were visibly confirmed at 3 days after plating. Around 4 to 5 days after plating, wells with colonies were fed by adding 100 μl of differentiation media with IL-7 (10 ng/ml), IL-15 (5 ng/ml), and DOX (1 μg/ml). All wells with colonies at day 3 were harvested, stained, and analyzed on a BDFortessa FACS analyzer with a high-throughput sampler on FACS Diva software (BD Biosciences). Colonies were scored for presence of erythrocytes (TER119^**+**^), platelets (CD41^**+**^), granulocytes (MAC-1^**+**^, GR1^**+**^), NK cells (NK1.1^**+**^), T cells (CD25^**+**^, LY6D^**+**^), and B cells (CD19^**+**^, LY6D^**+**^). LY6D is expressed on developing thymocytes and B cells and was useful as a second marker to identify T/B lymphocytes (Inlay et al., 2009). Colonies were scored as MegE if erythrocytes and/or platelets were detected, GM if granulocytes were detected, and L if NK, T, and/or B cells were detected. Colonies with MegE, GM, and L potential were scored as multipotent.

### Mouse Embryo Harvest and Cell Sorting

All animal procedures were approved by the International Animal Care and Use Committee and the Stanford Administrative Panel on Laboratory Animal Care. Matings of C57B6 mice were established and plugs checked in the mornings. Pregnant females were harvested in the mornings and embryos dissected immediately. Vitelline and umbilical vessels were typically harvested with the yolk sac, except where indicated. For the AGM harvest, the head, tails, feet, and fetal liver/heart were removed, and the remaining tissue was listed as AGM. Tissues were dissociated in 10 mg/ml Collagenase Type IV (Invitrogen 17104-019) for approximately 30 min to 1 hr, pipetted up and down and filtered in 70 micron mesh. Tissues were typically stained for 15 min on ice in staining media. See Table S1 for antibodies used. Cells were analyzed and/or sorted on a BD FACSAria using FACS Diva software. For clone sorting, a 100-micron nozzle was used, and cells were single sorted on “single cell” mode directly onto 96-well plates with ODT stroma. The authors highly recommend doing a single sort (as opposed to a double sort) due to substantial loss of rare cells upon the second sort. We also recommend avoiding the use of ACK lysis buffer prior to Ab staining, as it dramatically reduces the VE-cadherin signal.

### In Vivo Transplantation and Analysis

CD45.1^**+**^ CFP^**+**/**−**^ males were crossed with CD45.2^**+**^ females to produce CD45.1^**+**^ CD45.2^**+**^ embryos, half of which were CFP^**+**^ and half CFP^**−**^. CD11A^**−**^ KLS and CD11A^**+**^ KLS cells from e11.5 tissues were sorted and pooled such that CFP^**+**^ CD11A^**−**^ KLS cells were pooled with CFP^**−**^ CD11A^**+**^ KLS cells and vice versa. One hundred adult BM KLS cells (CD45.2^**+**^ CFP^**−**^) were also sorted from the mother and pooled with the sorted e11.5 KLS populations and then transplanted via the superficial facial vein into neonatal (days 1–3) NSG mice (CD45.1^**+**^) conditioned with 100 rads irradiation. Mice were bled at 4, 8, 12, and 15 weeks posttransplantation for analysis. Erythrocytes were lysed in ACK lysis buffer (150 mM NH_4_Cl, 10 mM KHCO_3_, 0.1 mM EDTA), and the remaining cells were stained with antibodies and analyzed on a BD FACSAria flow cytometer.

## Author Contributions

M.A.I., T.S., A.M., and I.L.W. designed experiments. M.A.I., T.S., and A.M. performed experiments. J.W.F., I.K.D., and J.S. contributed unpublished data, efforts, and tools. M.A.I. and T.S. wrote the manuscript, and I.L.W. edited the manuscript. All experiments were performed in the laboratory of I.L.W.

## Supplemental Information

Document S1. Supplemental Discussion, Supplemental Experimental Procedures, Figures S1–S7, and Table S1
