# Supplementary material for: Identification of Multipotent Progenitors that Emerge Prior to Hematopoietic Stem Cells in Embryonic Development
Source: Stem Cell Reports. 2014 Mar 20;2(4):457–72. doi: 10.1016/j.stemcr.2014.02.001 (PMC3986503; doi:10.1016/j.stemcr.2014.02.001)
Supplement: Document S1. Supplemental Discussion, Supplemental Experimental Procedures, Figures S1–S7, and Table S1 [file mmc1.pdf]

Stem Cell Reports, Volume *2*

Supplemental Information

Identification of the First Clonally Multipotent Hematopoietic Progenitors within the Mouse Embryo

Matthew A. Inlay, Thomas Serwold, Adriane Mosley, John W. Fathman, Ivan K. Dimov, Jun Seita, and Irving L. Weissman

INVENTORY OF SUPPLEMENTAL INFORMATION

Figure S1. Representative analyses of multipotent colonies (related to Figure 1).

Figure S2. Hematopoietic colony-forming potential in positive and negative fractions of single-marker stains of embryonic tissues (related to Figure 2).

Figure S3. Hematopoietic surface marker expression in the embryo (related to Figure 2).

Figure S4. Expression of HSC markers CD34, SlamF1, and Flk2 in YS, AGM, and FL from e9.5 to e11.5 (related to Figure 2).

Figure S5. Expression of endothelial and hematopoietic surface markers on CD11a$^-$ and CD11a$^+$ KLS cells (related to Figure 3).

Figure S6. The placenta contains CD11a$^-$ and CD11a$^+$ KLS cells (related to Figure 5).

Figure S7. Lineage tracing KLS cells *in vivo.*

Technical Discussion. A detailed discussion of the multipotency assay and surface markers used in the study.

Supplemental Experimental Procedures.

Table S1. Antibodies.

Supplemental References.

SUPPLEMENTAL FIGURES AND LEGENDS

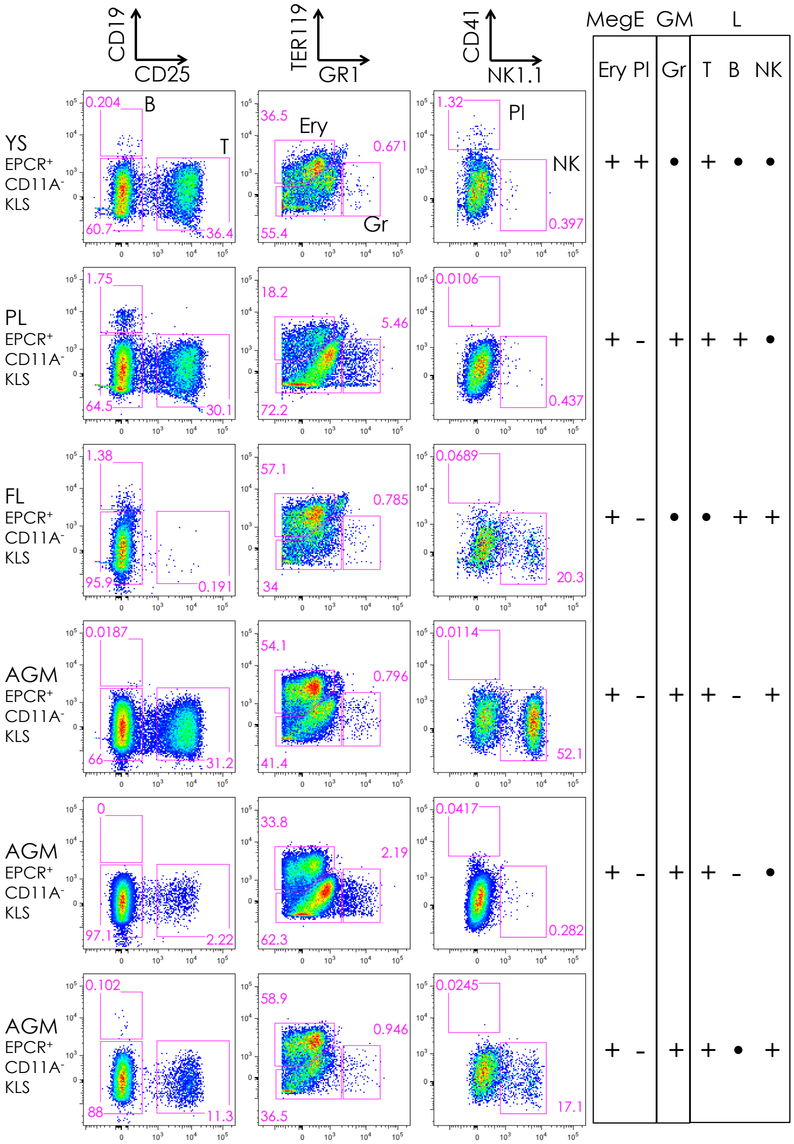

Figure S1. Representative analyses of multipotent colonies (related to Figure 1). A putative multipotent population from e11.5 tissues was clone sorted onto ODT stroma and analyzed at day 10 (see Fig. 1B for strategy). 6 representative colonies that were scored as multipotent are shown. The population clone sorted is described in Figure 5. The table on the right indicates how each colony was scored in each of the major hematopoietic lineages, including the Megakaryocyte/erythrocyte (MegE) branch composed of erythrocytes (TER119^{+}, Ery) and platelets (CD41^{+}, Pl), the granulocyte/monocyte (GM) branch composed of granulocytes (GR1^{+}, Gr), and the lymphoid (L) branch, composed of T cells (CD25^{+}, T), B cells (CD19^{+}, B), and natural killer cells (NK1.1^{+}, NK). Colonies were scored in each lineage as no readout ("-" < 0.1%), positive readout ("+", > 1%), and low readout ("**.**" < 1% but > 0.1%). Colonies with readout in at least one lineage for each major branch were scored as multipotent.

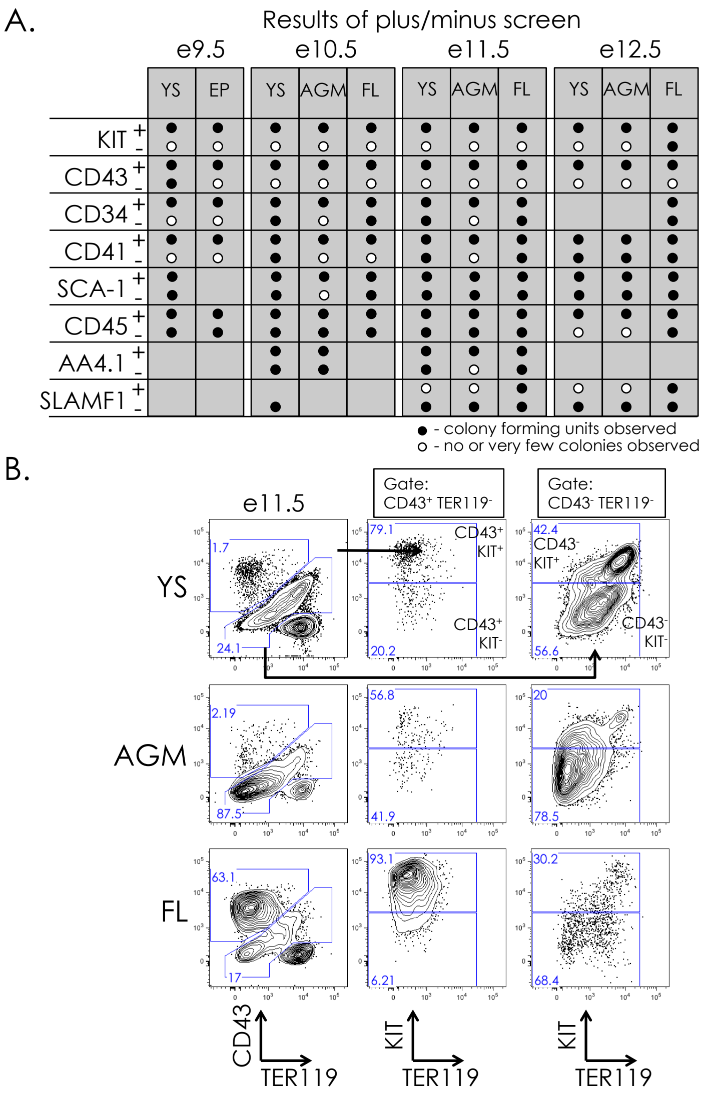

C.

hematopoietic colonies / # cells plated

	CD43⁺KIT⁺	CD43⁺KIT⁻	CD43⁻KIT⁺	CD43⁻KIT⁻
YS	51/200	1/167	1/5226	0/6288
AGM	13/200	0/789	0/10,000	0/25,000
FL	33/200	0/1000	1/2275	0/4135

Figure S2. Hematopoietic colony-forming potential in positive and negative fractions of single-marker stains of embryonic tissues (related to Figure 2). In order to identify markers that enrich or deplete for hematopoietic activity, we performed a plus/minus screen. We stained embryonic tissues from e9.5 to e12.5 with single markers, then sorted the entire positive and negative fraction of each, plated them on ODT stroma with cytokines, then at day 3 counted the number of colonies that formed. **A**) The table indicates whether we found colonies (black circle) or did not find colonies (white circle) forming in the positive (+) or negative (-) fraction. While for most markers, we identified colonies in both fractions, for two markers, CD43 and KIT, we only identified colonies in the + fraction, regardless of timepoint or tissue, with some exceptions. Thus, nearly all colony-forming activity is contained entirely within the KIT^{+} CD43^{+} fraction for all tissues and at all timepoints. **B**) Confirmation of plus/minus results for KIT and CD43 at e11.5. To confirm the finding from the plus/minus screen that all hematopoietic colony-forming activity was contained within the KIT^{+} CD43^{+} fraction, we sorted YS, AGM, and FL from e11.5 into CD43^{+} KIT^{+}, CD43^{+} KIT^{-}, CD43^{-} KIT^{+}, and CD43^{-} KIT^{-} fractions, and plated onto ODT stroma with cytokines. Only TER119^{-} cells (i.e. non-red blood cells) were sorted. **C**) The number of colonies observed at day 3 over the total number of cells plated for each tissue is shown. Note that only the CD43^{+} KIT^{+} fraction resulted in significant hematopoietic colonies at high frequency.

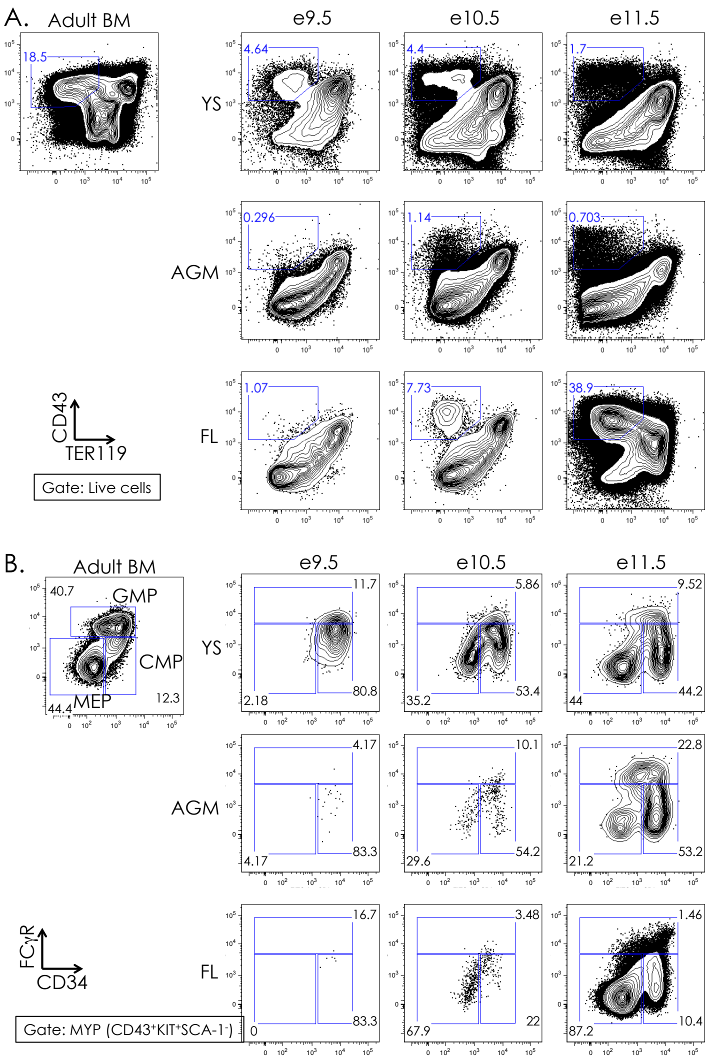

Figure S3. Hematopoietic surface marker expression in the embryo (related to Figure 2). **A**) CD43 expression in YS, AGM, and FL from e9.5 to e11.5. CD43 (y-axis) and TER119 (x-axis) expression for live cells is displayed. KIT and SCA-1 expression within the CD43^{+} TER119^{-} gate is shown in Figure 2. **B)** Myeloid progenitors (MYP) in YS, AGM, and FL from e9.5 to e11.5. FCγR (y-axis) vs. CD34 (x-axis) expression within the myeloid progenitor (MYP) gate (KIT^{+} Lin^{-} SCA-1^{-}, shown in Figure 2). Gates and percentages of phenotypic common myeloid progenitors (CMP), granulocyte-monocyte progenitors (GMP), and megakaryocyte-erythrocyte progenitors (MEP) are shown. At e9.5, a population that corresponds to CMP can be identified in YS MYP cells. At e10.5, MEP cells can be found in YS, AGM, and FL. Lastly, cells that correspond to GMP are observed at e11.5. We tested these populations for lineage potential on ODT stroma, and they have similar lineage output as their adult counterparts (data not shown). We did observe rare lymphocyte colonies arising from the CMP population at e11.5. IL7Rα is a marker for CLP, but didn't become expressed until e12.5. Without IL7Rα expression, CLP would fall within the CMP gate, and thus we expect CMP to contain some CLP and thus some lymphocyte activity.

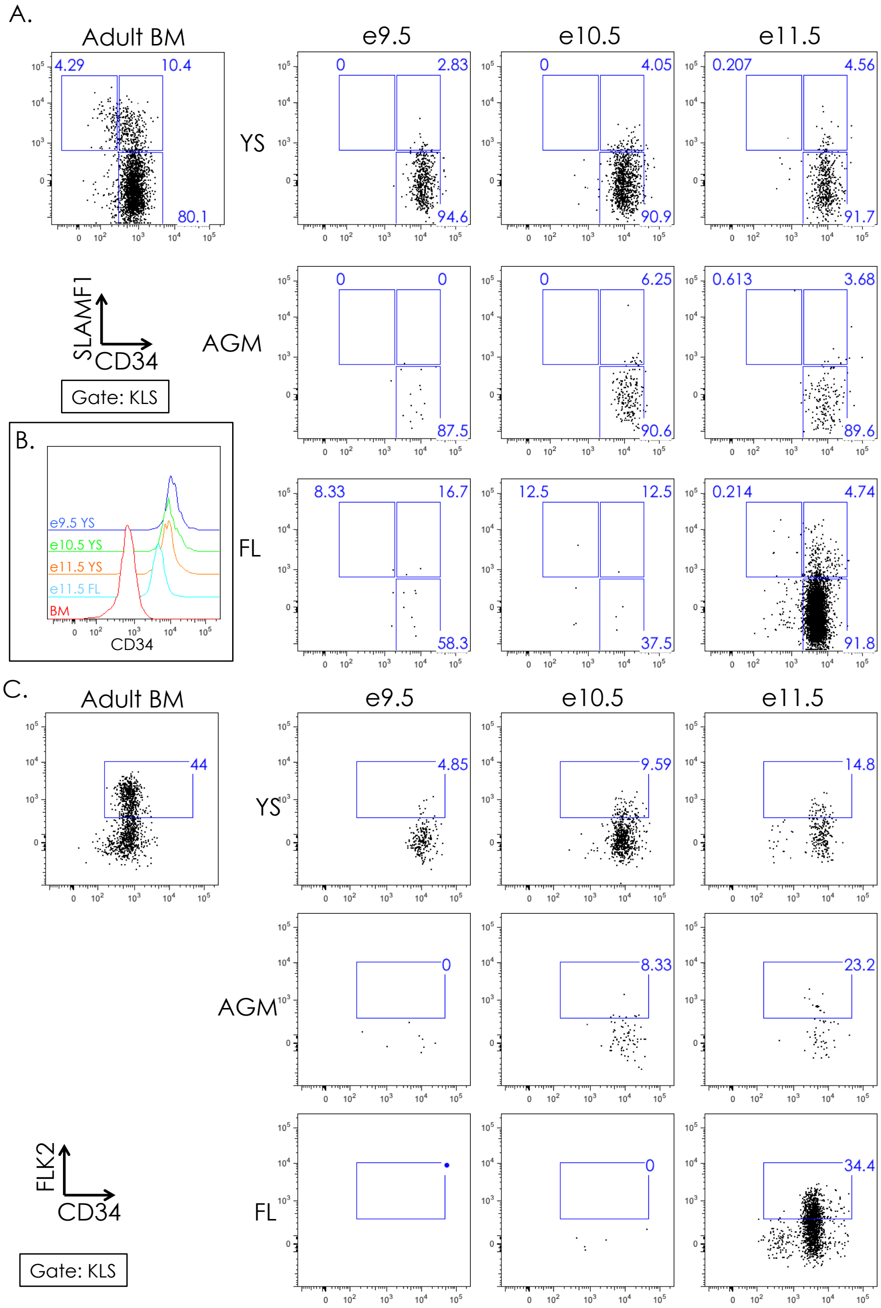

Figure S4. Expression of HSC markers CD34, SLAMF1, and FLK2 in YS, AGM, and FL from e9.5 to e11.5 (related to Figure 2).

Markers commonly used to subdivide adult BM KLS cells were analyzed for expression in embryonic KLS subsets. **A**) SLAMF1 and CD34 expression in KLS cells. Phenotypic adult HSCs are SLAMF1^{+} and CD34^{-}. Note that SLAMF1 does not upregulate until e11.5 and only in a small subset of YS and FL KLS cells. **B**) CD34 expression profile in KLS cells of YS (e9.5 blue, e10.5 green, e11.5 orange) and FL (e11.5 light blue) compared to BM (red). While CD34 expression decreases slowly from e9.5 to e11.5, all embryonic KLS cells express much higher levels of CD34 than adult BM KLS. **C**) FLK2 vs. CD34 expression in embryonic KLS. Only at e11.5 is FLK2 expression robust.

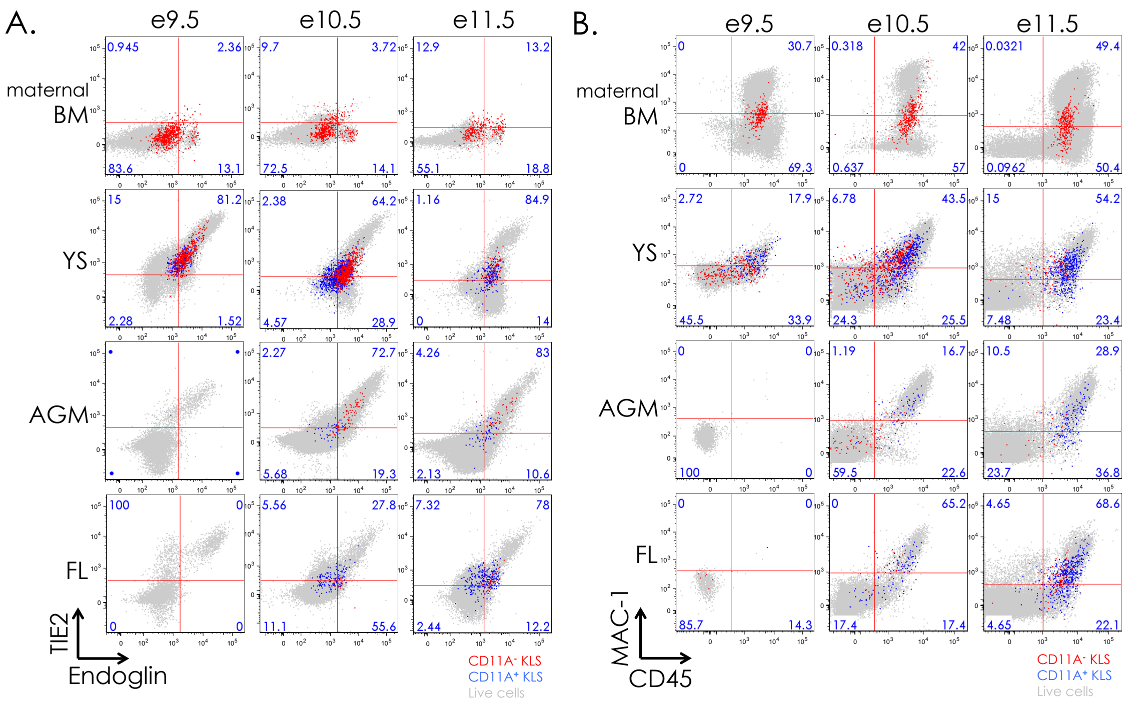

Figure S5. Expression of endothelial and hematopoietic surface markers on CD11A$^-$ and CD11A$^+$ KLS cells (Related to Figure 3).

In addition to CD11A and VE-CAD, we also examined the expression of several other surface markers on CD11A$^-$ and CD11A$^+$ KLS cells, including endothelial markers such as TIE2 and Endoglin (**A**), and hematopoietic markers such as MAC-1 and CD45 (**B**). **A**) Expression of TIE2 (y-axis) and Endoglin (x-axis) on CD11A$^-$ KLS (red) and CD11A$^+$ KLS (blue). Background expression of TIE2 and Endoglin among all live cells (grey) is shown. Percentages in quadrants are shown only for CD11A$^-$ KLS cells. For the BM, all KLS cells are shown in red. Our data suggest that CD11A$^-$ KLS cells generally have higher expression of TIE2 and Endoglin than CD11A$^+$ KLS cells for many tissues and timepoints. It should be noted that each timepoint was stained and analyzed in separate experiments, and thus differences across timepoints are not as significant as differences within a timepoint. **B**) Expression of MAC-1 (y-axis) and CD45 (x-axis) on CD11A$^-$ and CD11A$^+$ KLS cells. In contrast to TIE2 and Endoglin, the expression of these hematopoietic-specific surface markers appears to increase between CD11A$^-$ and CD11A$^+$ KLS fractions. Taken together, the results of these stains suggest that KLS cells initially express multiple endothelial markers (VE-CAD, TIE2, and Endoglin) and gradually downregulate these markers and upregulate hematopoietic-specific markers (CD11A, MAC-1, CD45).

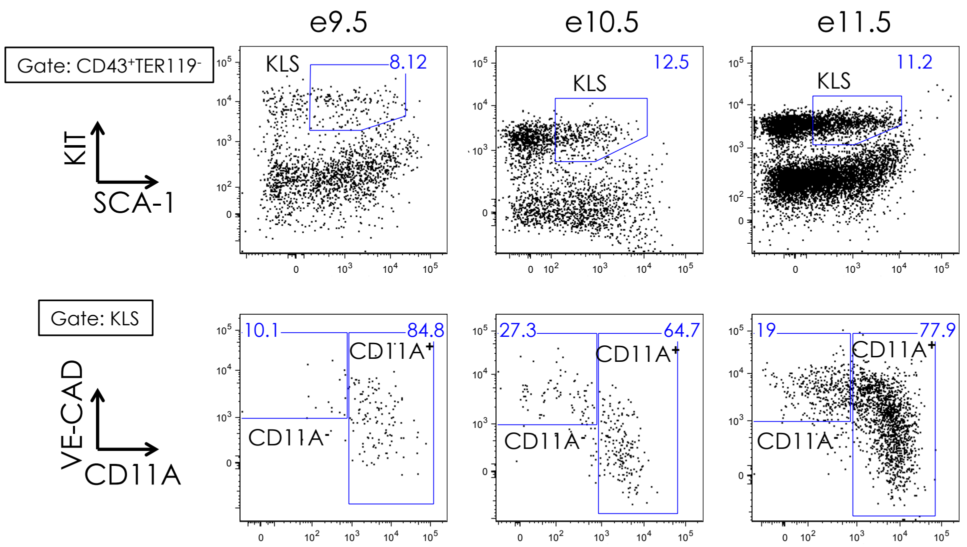

Figure S6. The placenta contains CD11A$^-$ and CD11A$^+$ KLS cells (related to Figure 5). FACS plots of e9.5, e10.5, and e11.5 PL. The top row is gated on live, CD43$^+$ TER119$^-$ cells, and the bottom row on KLS cells. Note that each timepoint was analyzed in separate experiments, and thus the staining intensities varied. While CD11A$^+$ KLS cells appear at e9.5 in the PL, CD11A$^-$ KLS cells do not appear until e10.5.

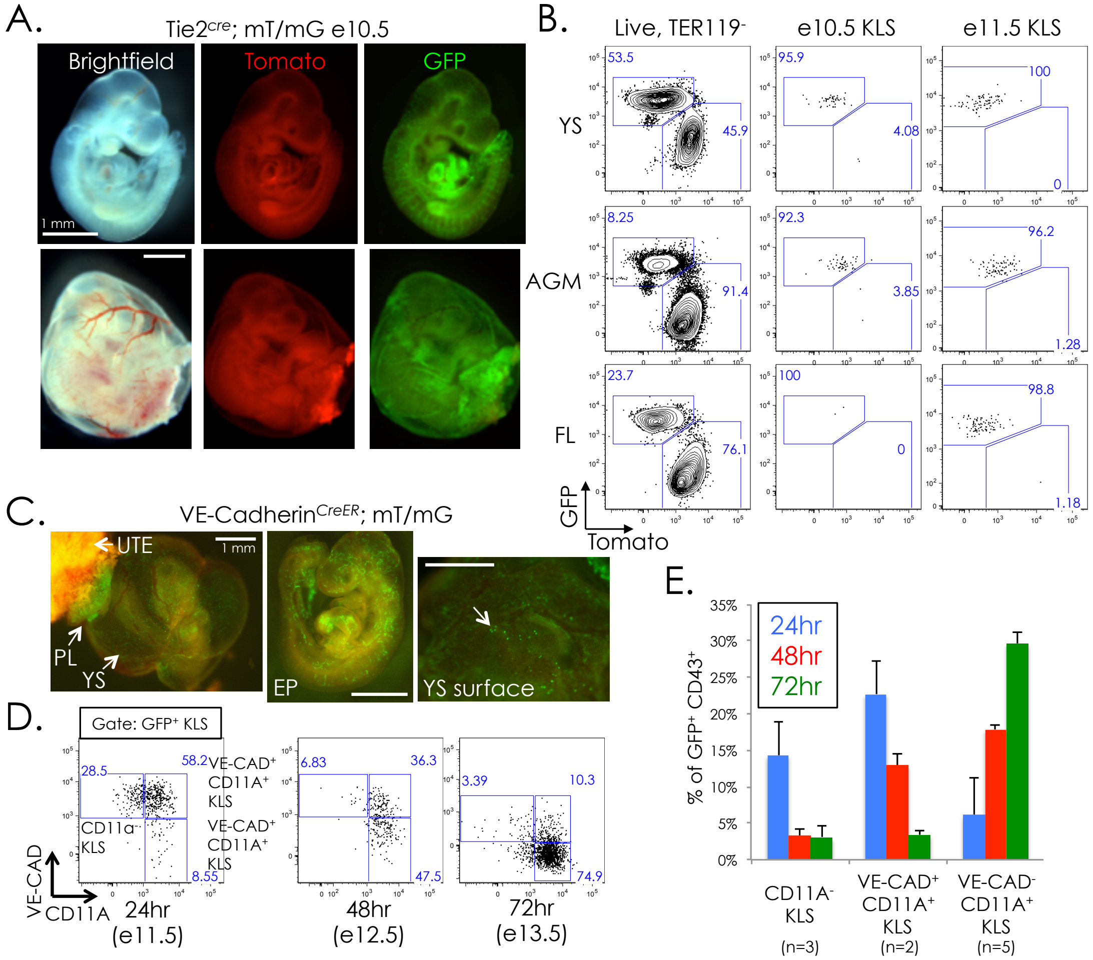

Figure S7. Lineage tracing KLS cells *in vivo*.
A) Visualization of reporter expression in Tie2^{cre} x mT/mG embryos at e10.5. Brightfield (left), Tomato (middle), and GFP (right) expression in the embryo proper alone (top row) and with the YS (bottom row) is shown. Scale bar (white) is 1 mm. **B)** Tomato vs. GFP expression is shown for all live, TER119^{-} cells (left column) compared to e10.5 KLS (middle column), and e11.5 KLS (right column) for YS, AGM, and FL. Nearly all KLS cells are GFP^{+}, indicating that they all originated from TIE2^{+} precursors. **C-E)** VE-Cadherin^{CreER} labeling of KLS cells. **C)** Visualization of GFP reporter expression in VE-Cadherin^{CreER} x mT/mG embryos at e9.5. Tamoxifen was injected at e8.5. Background orange color represents bleed-over from Tomato^{+} cells in the GFP channel. Left: Whole conceptus, including uterine tissue (UTE), placenta (PL) and YS. Middle: Embryo proper (EP). Right: YS surface. Arrow indicates punctate GFP^{+} cells, likely hematopoietic. Scale bar (white) is 1 mm. **D)** Analysis of GFP^{+} cells in the FL of VE-Cadherin^{CreER} x mT/mG embryos. Tamoxifen was injected at e10.5, and embryos harvested 24 hours (left), 48 hours (middle), or 72 hours later (right). Only GFP^{+} KLS cells are shown. **E)** Distribution of GFP^{+} KLS cells 24, 48, and 72 hours after tamoxifen injection. Percentage for each population is out of total CD43^{+} GFP^{+} cells. Our results show that percentage of GFP^{+} CD11A^{-} KLS and VE-CAD^{+} CD11A^{+} KLS cells decreases over time, and VE-CAD^{-} CD11A^{+} KLS cells increase, consistent with the notion that the VE-CAD^{+} KLS populations can give rise to VE-CAD^{-} CD11A^{+} KLS. Error bars are SD.

TECHNICAL DISCUSSION

Multipotency assay as a tool to identify definitive hematopoietic wave-initiating cells
In this study, we examined when multipotency arises in embryonic development, and used this property as a way to identify populations that may have the potential to give rise to fully functional adult-type HSCs. To accomplish this, we developed an *in vitro* assay to identify clonally multipotent populations. While our multipotency assay can reveal 8 major hematopoietic lineages, we rarely observed all 8 lineages simultaneously produced by a single cell. When we tested this assay on adult BM KLS, a population known to contain all multipotent cells, we found that only 15% of colonies contained lineages representing all three major hematopoietic branches (MegE, GM, L). It is likely that this assay underestimates the true number of multipotent cells in a given population. Furthermore, when we subdivided KLS into HSCs and 3 MPP populations, only HSCs gave multipotent readout, suggesting that more downstream multipotent progenitors are even less likely to be revealed as multipotent in this assay. The large number of cytokines and signals we utilize likely drives some multipotent cells to immediately commit to a lineage, and thus obscures their *in vivo* lineage potential. In addition, the lineages produced are in competition with one another for resources, such that as one lineage expands, it can suppress other lineages by reducing the available nutrients or growth factors or simply by overwhelming the well with numbers. Thus, any skewing of lineages produced by a particular cell may be a byproduct of the culture conditions and not an inherent lineage bias within that cell. For example, the day of *Dll1* induction by addition of DOX could greatly impact the ratio of T cells to B cells produced. Because of the variation inherent in this assay, we scored colonies as multipotent if they produced lineages representing each of the three major hematopoietic branches (MegE, GM, and L), even if other lineages were absent or low. For example, if a colony produced erythrocytes, granulocytes, and T cells, we scored this colony as multipotent even though platelets, B cells, and NK cells were not detected. The ability of a single cell to simulateously give rise to all three major branches is a property extremely difficult to reveal *in vitro* or *in vivo*, and thus we feel this assay is an important contribution to this field. While these caveats are important to note, this assay can still reveal multilineage potential in individual cells that are robustly multipotent, making our assay uniquely suited to identifying the populations that give rise to definitive hematopoietic waves.

Multipotency prior to e9.5
We find evidence of CD11A$^-$ KLS cells as early as e8.5, although we remain unable to produce lymphocytes from this developmental stage in our assay system. Others have reported B1 cell potential at e8.5 (Godin et al., 1993), but our assay has not revealed any lymphoid potential at that stage, even when we cultured whole unsorted tissues (data not shown).

CD11A$^-$ KLS in the placenta
The placenta is an important site of embryonic hematopoiesis, and likely produces HSCs and their precursors independently of other sites (Mikkola et al., 2005). We examined the placenta using our marker panel and identified CD11A$^-$/CD11A$^+$ KLS cells (Fig. 5, S6). However, the surface marker pattern in the placenta differed greatly from other

tissues, and many of the markers, including SCA-1, CD41, and EPCR had distinct patterns, resulting in populations that exist only in the placenta. While we identified multipotent cells within PL CD11A$^-$ KLS, it remains unclear whether there are other multipotent populations in the PL, and how the absolute number of multipotent cells in the placenta compares to other tissues.

CD11A$^-$ KLS in the vitelline and umbilical vessels, and the blood

When dissecting embryonic tissues, we erred on the side of taking too much tissue, in order to avoid losing important cells. For our AGM dissections, we took the entire caudal half of the embryo, after removing the FL, heart, gut, head, tail, and legs. Thus, it is possible there are fewer CD11A$^-$ KLS cells in the dorsal aorta than we estimate, but it is highly unlikely there are more. For our YS dissections, we often included portions of the umbilical and vitelline vessels, which have been shown to contain HSCs (de Bruijn et al., 2000). While the umbilical vessels, which arise from the allantois, are extra-embryonic in origin, the vitelline (omphalomesenteric) artery is considered intra-embryonic in origin. When we separated the vitelline and umbilical vessels, we found CD11A$^-$ KLS cells within them, but the bulk of CD11A$^-$ KLS remained in the YS (data not shown). When harvesting tissues, we also harvested the blood within them. Thus, it is possible that many CD11A$^-$ KLS are actively trafficking in the blood. However, visual examination of the GFP-labeled cells on the surface of the YS in VE-Cadherin^{CreER} x mT/mG embryos revealed that nearly all labeled cells were attached to the YS, and not in circulation (Fig. 6C). This suggests that VE-CAD$^+$ hematopoietic cells remain tightly bound to the vasculature. Despite these caveats, we feel our data indicates that the YS contains substantial numbers of definitive multipotent cells from e9.5 to e11.5.

Multipotency shifts to the FL

By e11.5, the FL contained by far the most hematopoietic progenitors as assayed by colony-forming ability, around 50,000 per FL (Fig. 4A). However, by our estimates the number of multipotent cells is substantially lower, around 30 per FL. It is likely that multiple populations infuse into the FL from e10.5 to e11.5, including CD11A$^+$ KLS, MYP, and other downstream progenitors. Thus, the FL is likely a co-mixture of cells representing multiple hematopoietic waves. When analyzing unsorted FL tissues plated onto ODT stroma, we observed the vast majority of colonies to be small erythroid colonies (data not shown), and the largest KIT$^+$ population in the FL at e11.5 are phenotypically similar to MEP (KIT$^+$ SCA-1$^-$ CD34$^-$, Fig. S3B). The FL is a hematopoietic niche that induces a robust expansion of hematopoietic cells. At e12.5, we observe a shift in the surface phenotype of multipotent cells, as multipotency is detected in the CD11A$^+$ KLS population, and our absolute number estimates suggest that at e12.5, the CD11A$^+$ KLS population contains the majority of multipotent cells. It remains unclear whether these multipotent CD11A$^+$ KLS cells derive from CD11A$^-$ KLS cells that upregulated CD11A, or whether existing CD11A$^+$ KLS cells mature and acquire multilineage potential. Based on our numbers, there do not appear to be sufficient numbers of CD11A$^-$ KLS cells to account for the increase in multipotent CD11A$^+$ KLS at e12.5, which suggests that many CD11A$^+$ KLS cells acquire multilineage potential.

Most embryonic KLS cells are not multipotent

In adult BM, the KLS population contains all multipotent stem and progenitor cells. However, in the embryo we find that only the CD11A$^-$ KLS subset contains the multipotent cells from e9.5 to e11.5. As the majority of KLS cells express CD11A and fall within the CD11A$^+$ KLS gate, this means that the majority of KLS cells in the embryo are not multipotent, and are likely a mixture of downstream lineage committed progenitors like CMP and CLP, or possibly primitive progenitors from an earlier wave. While most CD11A$^+$ KLS cells can produce all lineages at the population level, only by examining lineage potential at the single cell level could we reveal the limitation of the CD11A$^+$ KLS population, validating the utility of our clonal multipotency assay to reveal multilineage potential in individual cells.

It has long been known that the surface markers that define adult HSCs and downstream progenitors are not conserved in embryonic development, but it has been difficult to address this. Because there are multiple waves of hematopoiesis, originating from distinct sites, but all overlapping in a common circulatory system, it has been difficult to identify and isolate the key populations which represent the cells that could eventually become HSCs. Any attempt to molecularly characterize populations of cells requires first a careful characterization of the functional properties of those cells. Importantly, our work reveals that prior to e12.5, only a small fraction of KIT$^+$SCA-1$^+$ population contains multipotent cells. Thus, microarrays or RNA sequencing databases containing KIT$^+$SCA-1$^+$ cells may be contaminated with downstream lineage-committed cells, and thus attempts to identify the key genes or pathways involved in the maturation of HSCs from an earlier cell type may be partially skewed.

CD41 vs CD43 as a hematopoietic marker

CD41 is used as a platelet marker in adult hematopoiesis, or a marker of megakaryocyte progenitors (Pronk et al., 2007), but is also a marker of early hematopoietic cells in embryonic development (Corbel and Salaun, 2002; Ferkowicz et al., 2003; Mikkola et al., 2003; Mitjavila-Garcia et al., 2002). We compared the utility of CD41 compared to CD43, and indeed CD41 is expressed on hematopoietic cells (data not shown). However, its expression began to downregulate in the stem/progenitor populations and increased in platelet precursors from e9.5 to e11.5. It has already been reported that CD41^{low} cells contain the relevant hematopoietic cells (Robin et al., 2011; Rybtsov et al., 2011). Thus, while CD41 is highly expressed at the earliest stages of hematopoietic specification, its gradual downregulation over time made it less reliable as a hematopoietic marker in our opinion than CD43. However, CD41 was highly expressed in e9.5 hematopoietic cells, and may serve as a better hematopoietic marker than CD43 at this specific timepoint.

CD45

CD45 has long been considered the definitive pan-hematopoietic marker, expressed on all hematopoietic cells except for mature erythrocytes and platelets. However, the earliest hematopoietic cells in the embryo are CD45$^-$, and most CD11A$^-$ KLS cells at e9.5 and e10.5 are also CD45$^-$ (Fig. S5). However, CD45 is expressed on all CD11A$^+$ KLS and downstream cells, suggesting that it upregulates as CD11A$^-$ KLS cells differentiate. When we examined e11.5 tissues, CD45 was expressed on CD11A$^-$ KLS, indicating that

CD11A$^-$ KLS cells slowly upregulate this marker from e9.5 to e11.5. CD45 upregulation likely occurs after CD11A$^-$ KLS cells diverge from their putative hemato-endothelial origins, and is possibly a reflection on how long ago that divergence occurred. CD45 and CD41 have essentially opposite expression changes over time. Initially, all hematopoietic progenitors are CD41$^+$ but downregulate over time, whereas these same progenitors are initially CD45$^-$, but upregulate it over time. This is primarily why we prefer CD43 as a hematopoietic marker, as its expression in embryonic hematopoietic progenitors remains relatively uniform during these stages.

Utility of EPCR as a marker of multipotent cells
In adults, EPCR is specifically expressed on HSCs and FLK2$^-$ MPPs, and is becoming a useful marker for HSC isolation (Balazs et al., 2006; Benz et al., 2012). We also explored whether EPCR could enrich for multipotent cells. Our data suggest that EPCR can partially enrich for multipotent cells. Given that VE-Cadherin slowly downregulates over time in CD11A$^+$ KLS, as in e11.5 and e12.5 FL KLS, EPCR becomes a useful marker in separating out multipotent cells from downstream lineage-committed progenitors. It is possible that multipotent cells that are EPCR$^-$ may represent a population downstream of EPCR$^+$ cells, and that EPCR$^-$ KLS cells represent the embryonic equivalent of MPP, whereas EPCR$^+$ KLS cells are precursors to HSCs. In support of this, Iwasaki et al. observed that in e12.5 FL, EPCR$^+$ KLS cells could engraft long-term in adult mice, but EPCR$^-$ KLS could not (Iwasaki et al., 2010).

SCA-1 antibodies to sort multipotent cells
Others have reported that SCA-1 (Ly-6A) surface expression in mouse embryos is not robust enough to serve as a reliable marker to identify and isolate SCA-1$^+$ cells, and have instead developed a SCA-1-GFP transgenic reporter for detection of SCA-1 expression in the embryonic development (de Bruijn et al., 2002; Ma et al., 2002). We have found that SCA-1 antibodies are suitable to detect SCA-1 expression in the embryo, although the expression was significantly less than that in BM, rendering many SCA-1-fluorochrome conjugations unsuitable for sorting SCA-1$^+$ cells. We recommend testing conjugations on adult BM cells and only using the brightest conjugates. In our hands, only SCA-1-PE and SCA-1-PECy7 conjugations were bright enough to use for our experiments. Please refer to supplemental Table S1 for catalog information on all antibodies used in these experiments.

SUPPLEMENTAL EXPERIMENTAL PROCECURES

Generation of inducible OP9-DL1 (ODT) stroma

Two lentiviral constructs were generated from the pCDH-EF1α (System Biosciences, Cat no. CD521A) backbone. In construct C329 (also described in (Ardehali et al., 2011), the tet-ON transactivator rtTA3 was inserted into pCDH-EF1α. The GFP from pCDH-EF1α was replaced with turboRFP. In construct C388, mouse *Dll1* (Open Biosystems cat. # MMM1013-9202259) was inserted into a pCDH construct (C298, also described in (Ardehali et al., 2011)) where the EF1α promoter was replaced by the tet-responsive element (TRE). OP9 stroma was infected first with C329, and transduced cells were sorted by RFP expression. RFP^+ cells were then infected with C388, and DLL1-GFP expression was induced by addition of doxycycline (DOX, 1 μg/mL), and GFP^+ $DLL1^+$ cells were sorted. The RFP^+ GFP^+ cells were then cultured in the absence of DOX until GFP expression returned to baseline, then transduced OP9 cells were resorted to remove cells with high background GFP/DLL1 expression. It should be noted that the final cell line, ODT, is polyclonal.

$Tie2^{Cre}$ and VE-$Cadherin^{CreER}$ lineage tracing

$Tie2^{Cre}$ homozygous males were crossed to mT/mG homozygous females. For VE-$Cadherin^{CreER}$ experiments, males homozygous for VE-$Cadherin^{CreER}$ and mT/mG were crossed to Wt females. 4 mg of tamoxifen (Sigma T-5648) was injected (two injections of 100 μL of 20 mg/mL intraperitoneally). To prepare tamoxifen solution, tamoxifen was dissolved in corn oil (Sigma C8267) at 20 mg/mL, pipetted vigorously, and warmed for 20' in a 37° C water bath, with occasional mixing, then briefly centrifuged to remove undissolved particles. GFP fluorescence was visualized and photographed on a Leica M205 FA fluorescent dissecting scope with Leica Application Suite v4.1 software.

Absolute cell number counts

To estimate the absolute cell numbers, a defined number of accudrop beads (BD Biosciences 34529) was added to each sample (e.g. 10,000 beads per sample). Accudrop beads are easily identifiable as extremely bright in Cy7 channels (e.g. PECy7, APCCy7). The number of recovered beads was used to estimate the fraction of sample present in each FACS file (e.g. if 5000 beads were recovered out of 10,000 added, then ½ the sample was present in the FACS file).

Table S1. Antibodies

Antigen	Clone	Conjugate	Source	Catalog Number
B220	RA3-6B2	FITC	eBioscience	11-0452-81
CD3	17A2	PerCP-eFluor710	eBioscience	46-0032-80
CD11C	N418	APC-eFluor 780	eBioscience	47-0114-80
CD19	1D3	PECy5.5	eBioscience	discontinued
	ebio1d3	APC	eBioscience	17-0193-80
CD25	PC61	APC	Weissman	n/a
	PC61	APCCy7	Biolegend	102025
CD27	LG.7F9	APC	eBioscience	17-0271-81
CD34	RAM34	FITC	eBioscience	11-0341-81
CD41	MWreg30	PE	Biolegend	133905
	MWreg30	PerCP-eFluor710	eBioscience	46-0411-80
	MWreg30	APC-eFluor780	eBioscience	47-0411-80
CD43	eBioR2/60	PE	eBioscience	12-0431-81
	S7	APC	BD Bioscience	560663
	eBioR2/60	Biotin	eBioscience	13-0431-81
CD45.1	A20.1.7	Alexa680	Weissman	n/a
	A20	PECy7	Biolegend	110729
CD45.2	104	FITC	Biolegend	109805
KIT (CD117)	2B8	PECy7	eBioscience	25-1171-81
	2B8	Alexa780	eBioscience	47-1171-80
	2B8	Brilliant Violet 42	Biolegend	105827
Endoglin (CD105)	MJ7/18	eFluor-450	eBioscience	48-1051-80
EPCR (CD201)	eBio1560	PerCP-eFluor710	eBioscience	46-2012-80
FCGR (CD16/32)	93	APC	eBioscience	17-0161-81
	93	APCCy7	Biolegend	101327
FLK2	A2F10	PE	eBioscience	12-1351-83
	A2F10	Biotin	eBioscience	13-1351-81
GR1	RB6-8C5	PECy5	eBioscience	15-5931-81
	RB6-8C5	Alexa700	Biolegend	108421
	RB6-8C5	eFluor450	eBioscience	48-5931-80
LFA-1 (CD11A)	M17/4	PE	Biolegend	101107
	M17/4	Biotin	Biolegend	101103
LY6D	49H4.3	Alexa680	Weissman	n/a
MAC-1	M1/70	FITC	eBioscience	11-0112-82
	M1/70	PECy5	eBioscience	15-0112-81
	M1/70	Pacific Blue	Biolegend	101224
NK1.1	PK136	PE	eBioscience	12-5941-81
	PK136	PECy7	eBioscience	25-5941-81
SCA-1	D7	PE	Biolegend	122507
	e13-161.7	PECy7	Biolegend	122513
	D7	PECy5.5	eBioscience	35-5981-82
SLAMF1 (CD150)	mShad150	PerCP-eFluor710	eBioscience	46-1502-82
	TC15-12F12.2	PECy7	Biolegend	115913
	TC15-12F12.2	Brilliant Violet 42	Biolegend	115925
TIE2 (CD202b)	TEK4	Biotin	eBioscience	13-5987-81
TER119	TER119	PECy5	eBioscience	15-5921-81
	TER119	PECy7	eBioscience	25-5921-81
THY1.1	19XE5	PE	Weissman	n/a
	HIS51	Biotin	eBioscience	13-0900-81
VE-Cadherin (CD144)	BV13	Biotin	eBioscience	138008
streptavidin	n/a	Qdot605	Invitrogen	Q10101MP

SUPPLEMENTAL REFERENCES

Ardehali, R., Inlay, M.A., Ali, S.R., Tang, C., Drukker, M., and Weissman, I.L. (2011). Overexpression of BCL2 enhances survival of human embryonic stem cells during stress and obviates the requirement for serum factors. Proc Natl Acad Sci U S A *108*, 3282-3287.

Balazs, A.B., Fabian, A.J., Esmon, C.T., and Mulligan, R.C. (2006). Endothelial protein C receptor (CD201) explicitly identifies hematopoietic stem cells in murine bone marrow. Blood *107*, 2317-2321.

Benz, C., Copley, M.R., Kent, D.G., Wohrer, S., Cortes, A., Aghaeepour, N., Ma, E., Mader, H., Rowe, K., Day, C.*, et al.* (2012). Hematopoietic stem cell subtypes expand differentially during development and display distinct lymphopoietic programs. Cell Stem Cell *10*, 273-283.

Corbel, C., and Salaun, J. (2002). AlphaIIb integrin expression during development of the murine hemopoietic system. Dev Biol *243*, 301-311.

de Bruijn, M.F., Ma, X., Robin, C., Ottersbach, K., Sanchez, M.J., and Dzierzak, E. (2002). Hematopoietic stem cells localize to the endothelial cell layer in the midgestation mouse aorta. Immunity *16*, 673-683.

de Bruijn, M.F., Speck, N.A., Peeters, M.C., and Dzierzak, E. (2000). Definitive hematopoietic stem cells first develop within the major arterial regions of the mouse embryo. Embo J *19*, 2465-2474.

Ferkowicz, M.J., Starr, M., Xie, X., Li, W., Johnson, S.A., Shelley, W.C., Morrison, P.R., and Yoder, M.C. (2003). CD41 expression defines the onset of primitive and definitive hematopoiesis in the murine embryo. Development *130*, 4393-4403.

Godin, I.E., Garcia-Porrero, J.A., Coutinho, A., Dieterlen-Lievre, F., and Marcos, M.A. (1993). Para-aortic splanchnopleura from early mouse embryos contains B1a cell progenitors. Nature *364*, 67-70.

Iwasaki, H., Arai, F., Kubota, Y., Dahl, M., and Suda, T. (2010). Endothelial protein C receptor-expressing hematopoietic stem cells reside in the perisinusoidal niche in fetal liver. Blood *116*, 544-553.

Ma, X., Robin, C., Ottersbach, K., and Dzierzak, E. (2002). The Ly-6A (Sca-1) GFP transgene is expressed in all adult mouse hematopoietic stem cells. Stem Cells *20*, 514-521.

Mikkola, H.K., Fujiwara, Y., Schlaeger, T.M., Traver, D., and Orkin, S.H. (2003). Expression of CD41 marks the initiation of definitive hematopoiesis in the mouse embryo. Blood *101*, 508-516.

Mikkola, H.K., Gekas, C., Orkin, S.H., and Dieterlen-Lievre, F. (2005). Placenta as a site for hematopoietic stem cell development. Exp Hematol *33*, 1048-1054.

Mitjavila-Garcia, M.T., Cailleret, M., Godin, I., Nogueira, M.M., Cohen-Solal, K., Schiavon, V., Lecluse, Y., Le Pesteur, F., Lagrue, A.H., and Vainchenker, W. (2002). Expression of CD41 on hematopoietic progenitors derived from embryonic hematopoietic cells. Development *129*, 2003-2013.

Pronk, C.J., Rossi, D.J., Mansson, R., Attema, J.L., Norddahl, G.L., Chan, C.K., Sigvardsson, M., Weissman, I.L., and Bryder, D. (2007). Elucidation of the phenotypic, functional, and molecular topography of a myeloerythroid progenitor cell hierarchy. Cell Stem Cell *1*, 428-442.

Robin, C., Ottersbach, K., Boisset, J.C., Oziemlak, A., and Dzierzak, E. (2011). CD41 is developmentally regulated and differentially expressed on mouse hematopoietic stem cells. Blood *117*, 5088-5091.

Rybtsov, S., Sobiesiak, M., Taoudi, S., Souilhol, C., Senserrich, J., Liakhovitskaia, A., Ivanovs, A., Frampton, J., Zhao, S., and Medvinsky, A. (2011). Hierarchical organization and early hematopoietic specification of the developing HSC lineage in the AGM region. J Exp Med *208*, 1305-1315.
